# Identification and characterization of the *Onchocerca volvulus* Excretory Secretory Product Ov28CRP, a putative GM2 activator protein

**DOI:** 10.1371/journal.pntd.0007591

**Published:** 2019-07-22

**Authors:** Ferdinand Ngale Njume, Stephen Mbigha Ghogomu, Robert Adamu Shey, Lea Olive Tchouate Gainkam, Philippe Poelvoorde, Perrine Humblet, Joseph Kamgno, Annie Robert, Leon Mutesa, Christophe Lelubre, Evelina Edelweiss, Arnaud Poterszman, Susi Anheuser, Luc Vanhamme, Jacob Souopgui

**Affiliations:** 1 Department of Molecular Biology, Institute of Biology and Molecular Medicine, IBMM, Université Libre de Bruxelles, Gosselies, Belgium; 2 Molecular and Cell Biology Laboratory, Biotechnology Unit, University of Buea, Buea, Cameroon; 3 École de santé publique, Université Libre de Bruxelles, Bruxelles, Belgium; 4 Department of Epidemiology, Centre for research on filariasis and other tropical diseases, Yaounde, Cameroon; 5 Faculté de santé publique, Institut de recherche expérimentale et clinique, Pôle d'épidémiologie et biostatistique, Université Catholique de Louvain, Clos Chapelle-aux-champs, Woluwe-Saint-Lambert, Belgium; 6 Center for Human Genetics, College of Medicine and Health Sciences, University of Rwanda, Kigali, Rwanda; 7 Laboratoire de Médecine Expérimentale, Université Libre de Bruxelles (ULB)—Unité 222, CHU Charleroi (Hôpital André Vésale), Rue de Gozée, Montigny-Le-Tilleul, Belgium; 8 Department of Integrated Structural Biology, Institut de Génétique et de Biologie Moléculaire et Cellulaire (IGBMC), Centre National de la Recherche Scientifique, UMR7104, Illkirch, France; 9 Department of Integrated Structural Biology, Institut de Génétique et de Biologie Moléculaire et Cellulaire (IGBMC), Institut National de la Santé et de la Recherche Médicale, UMR7104, Illkirch, France; 10 Department of Integrated Structural Biology, Institut de Génétique et de Biologie Moléculaire et Cellulaire (IGBMC), Université de Strasbourg, UMR7104, Illkirch, France; 11 LIMES Institute, Membrane Biology & Lipid Biochemistry Unit, c/o Kekulé-Institut für Organische Chemie und Biochemie, Universität Bonn, Bonn, Germany; Faculty of Science, Ain Shams University (ASU), EGYPT

## Abstract

*Onchocerca volvulus* is the nematode pathogen responsible for human onchocerciasis also known as “River blindness”, a neglected tropical disease that affects up to 18 million people worldwide. Helminths Excretory Secretory Products (ESPs) constitute a rich repertoire of molecules that can be exploited for host-parasite relationship, diagnosis and vaccine studies. Here, we report, using a range of molecular techniques including PCR, western blot, recombinant DNA technology, ELISA, high performance thin-layer chromatography and mass spectrometry that the 28 KDa cysteine-rich protein (Ov28CRP) is a reliable component of the *O*. *volvulus* ESPs to address the biology of this parasite. We showed that (1) Ov28CRP is a putative ganglioside GM2 Activator Protein (GM2AP) conserved in nematode; (2) OvGM2AP gene is transcriptionally activated in all investigated stages of the parasitic life cycle, including larval and adult stages; (3) The full-length OvGM2AP was detected in *in-vitro O*. *volvulus* ESPs of adult and larval stages; (4) the mass expressed and purified recombinant OvGM2AP purified from insect cell culture medium was found to be glycosylated at asparagine 173 and lacked N-terminal signal peptide sequence; (5) the recombinant OvGM2AP discriminated serum samples of infected and uninfected individuals; (6) OvGM2AP competitively inhibits MUG degradation by recombinant β-hexosaminidase A but not MUGS, and could not hydrolyze the GM2 to GM3; (7) humoral immune responses to the recombinant OvGM2AP revealed a negative correlation with ivermectin treatment. Altogether, our findings suggest for the first time that OvGM2AP is an antigenic molecule whose biochemical and immunological features are important to gain more insight into our understanding of host-parasite relationship, as well as its function in parasite development at large.

## Author summary

Human onchocerciasis is a neglected tropical disease affecting millions in endemic tropical countries and is the world’s second leading cause of infectious blindness. *Onchocerca volvulus*, the causative agent of the disease is a tissue dwelling nematode diagnosed by identification of microfilaria larval stages in skin snip biopsies. The development of new tools for the management of the disease requires the characterization of more parasite antigens as about 44% of genes have no predicted function. In this research, efforts were made to characterize one such novel secretory protein of the parasite. We report that it is a putative orthologue of the human GM2 activator protein and evaluated its immune response in the context of disease diagnosis. Although we observed cross-reaction with *Loa loa* and other related nematodes, our data still supports further investigations towards a possible function of the protein in immunomodulation.

## Introduction

Human onchocerciasis also known as River Blindness is a tropical disease caused by the parasitic nematode *Onchocerca volvulus* and is the world’s second leading cause of infectious blindness after trachoma. The infective larval stages (L3) are transmitted through the bites of infective blackflies of the genus *Simulium*. They eventually give rise to adults that dwell in subcutaneous tissues where they can survive for about 15 years, with adult females hatching about 1600 microfilariae daily [1,2]. About 18 million people carry dermal *O*. *volvulus* microfilariae (Mf) worldwide with 99% of them living in Africa. Mfs are responsible for severe itching or dermatitis experienced by about 6.5 million people and blindness affecting 270,000. Current estimates hold that about 187 million people are at risk of being infected and about 1.1 million disability-adjusted life-years (DALYs) were lost in 2015 due to onchocerciasis [3–5].

Onchocerciasis remains a public health problem in endemic communities despite advances made by ivermectin treatment in reducing the burden of the disease. In fact, due to limitation in the different segments of the past and ongoing control programs, including the risks associated with loasis co-endemicity [6], ivermectin resistance reported in some endemic foci [7–9] and the logistic/financial burden involved in implementing the mass drug administration programs [10], it is increasingly accepted that ivermectin treatment alone will not be capable of supporting disease elimination. Moxidectin, a recently FDA approved microfilaricidal milbemycin macrocyclic lactone, ushers a glimmer of hope as it is reported to be more efficient with a low affinity to p-glycoprotein transporters [11] and a 20–43 days lifespan [12–15]. However, the effect of repeated treatment as well as treatment of *Loa loa* coinfected individuals still needs to be assessed for evaluation of long-term effects of moxidectin-based elimination programs [16]. Furthermore, existing diagnostic tools, mainly the skin snip test and the antibody based Ov16 test [17], suffer drawbacks hence requiring supplementary tests for post treatment surveillance [18]. The search for novel drugs, vaccines and diagnostic tools therefore remain imperative for efficient surveillance of disease elimination [19]. The characterization of novel putative targets in the *Onchocerca* genome will help to meet these concerns. Genomics, proteomics and bioinformatics studies of *Onchocerca* have been reported and have indeed provided insights into molecules that could be useful in the context of disease diagnosis, therapy or vaccine design [20–24]. However, most of these high throughput researches call for more studies to characterize these proteins on an individual basis.

Parasite Excretory Secretory Products (ESPs) constitute the set of molecules produced by the parasite into the host environment. Helminth ESPs are known to perform a wide range of functions including modulation of host immune response, leading to immune evasion by the parasite, remodeling of host tissue giving rise to nodule formation and alteration of host tissue nutritional status amongst others [25]. Furthermore, the immunomodulatory properties of helminth ESPs and their potential use to treat allergy and autoimmune diseases [26,27] as well as metabolic syndrome have been reported [28]. The exploitation of ESPs of *Onchocerca* in the search for novel drugs, vaccines, diagnostics as well as mediators of immune response have also been described [29–33]. Given that the *Onchocerca* nodule is known to be vascularized [34,35], such molecules could reach host circulation thereby supporting investigations towards the detection of *O*. *volvulus* ESPs in body fluids.

The ganglioside GM2 Activator Protein (GM2AP) of *O*. *volvulus* is one of such ESPs. In other species, GM2AP functions *in vivo* as an essential cofactor of N-acetylhexosaminidase A in degrading the ganglioside GM2 in the lysosome [36,37]. GM2AP acts as a biological detergent, solubilizes, binds and transports different lipids [37,38]. The human GM2AP is best studied; its crystal structure has been established and some functional roles attributed to its domain [39,40]. A nematode GM2AP was also identified (in *Trichinella pseudospiralis*) and its unusual characteristics reported. Contrary to known canonical GM2AP function, this parasite orthologue does not facilitate degradation of GM2 ganglioside by N-acetylhexosaminidase A, although it does inhibit phospholipase D activity. This was correlated to the absence of a domain implicated in binding to hexosaminidase A [41].

In the current study, we identified and characterized the *O*. *volvulus* GM2AP herein denoted OvGM2AP. Our findings on its expression during the parasite life cycle and on the humoral responses to recombinant OvGM2AP expressed and purified from insect cells suggest that OvGM2AP is an antigenic molecule whose biochemical and immunological features are important to gain more insight into our understanding of host-parasite relationship, and ultimately, to elucidate the function of this new GM2AP.

## Materials and methods

### Ethics statement

Ethical approval for this study was obtained from the Cameroonian National Ethics Committee for Health Research (N^o^ 2015/01/543/CE/CNERSH/SP) and administrative authorization was obtained from the Cameroonian Ministry of Public Health. A written consent was obtained from all participants employed in the study. For minors, the consent forms were validated by parents or guardians. Participation was entirely voluntary and individuals were free to opt out at their discretion without fear of community leaders or health practitioners. Ethical concerns working with rabbit are addressed by the GenScript Institutional Animal Care and Use Committee (IACUC), licence # SYXK(SU) 2008–0021. The IACUC is accredited by the AAALAC (approval date: 6/19/2009).

### *Onchocerca* parasite material

*O*. *volvulus* and *O*. *ochengi* parasite material were collected at different stages of the parasites. *O*. *volvulus* nodules were obtained from patients in the Kombone Health area of the South-Western Region of Cameroon by a trained medical doctor and *O*. *volvulus* worms were obtained from these nodules as previously described [42]. Briefly, average nodular masses were digested in 0.5 mg/ml collagenase for 9 h at 37 ^o^C shaking at 90 rpm after which the male and female worms were cultured in incomplete RPMI (Gibco, USA) supplemented with 0.25 mg/ml gentamycin and L-glutamine. Bloodless skin snips were also collected from the left and right knee of heavily infected individuals (microfilaria (Mf) load greater than 150 Mfs per mg of skin) and placed in incomplete culture medium for 12 h for Mf to emerge. The worms were pipetted and washed thrice with 20% percoll. L2 and L3 larval stages were obtained from infected and/or infective blackflies as described elsewhere [43]. Briefly, infected *Simulium* flies were captured and grown in the laboratory for seven days after which the thorax and head regions were excised to obtain L2s and L3s respectively.

Infected cattle skins containing *O*. *ochengi* nodules were obtained from the abattoir in Douala and immediately brought to the laboratory where *O*. *ochengi* worms were obtained from these nodules as previously described [44]. These infected cattle skins were selected following a search on available skins obtained after regular slaughtering and skinning procedures carried out at the abattoir.

Only motile worms were used for the extraction of RNA, protein crude extracts and ESPs. Protein crude extracts were obtained by crushing worm samples in lysis buffer (150 mM NaCl, 1.0% Triton X-100 and 50 mM Tris pH 8.0) supplemented with 1 mM Phenylmethylsulfonyl fluoride (PMSF). ESPs were obtained by culturing parasites *in vitro* using incomplete RPMI medium (Gibco, USA). After 16 h of culture of parasites (4 adult females per ml of RPMI medium, 50 adult males and 800 L3s per 300 μl of RPMI medium) *in vitro*, the medium was collected, quantified by Bradford assay and used for SDS-PAGE and western blot analyses.

### Serum samples

Following examination of participants involved in the study by trained medical personnel, blood was collected from patients living in the endemic region of Kombone Health Area of the Mbonge Health district, South Western Region, Cameroon. *O*. *volvulus* is known to be specific in this region at the exclusion of other filarial infections. These patients (OVS) were selected on the basis of an established presence of Mf in skin snip biopsies and/or presence of clinical manifestation of onchocerciasis. The clinical and demographic data of these patients are indicated in S1 Table.

Sera were obtained from blood samples by employing an established protocol [45], diluted 1:2 in glycerol and stored at -20 ^o^C. Sera obtained from individuals residing in an onchocerciasis hypo-endemic region (HES) of Huye, Rwanda, and from European subjects (ESC), were used as controls. Sera were also collected from individuals in Bandjoun (Bandjoun, Cameroon), a region which has been on constant ivermectin administration for over two decades. Sera were collected from individuals whose infection status had previously been well characterized [46]. *Loa loa* serum (LLS) samples were collected from the loiasis patients in the endemic regions of the Mvila division in the rain forest of the southern region of Cameroon in areas with high loiasis endemicity but <20% prevalence of onchocerciasis and with no ongoing CDTI programs. These subjects had been previously recruited for a study on the effects of albendazole on *L*. *loa* microfilariae [47]. These samples were obtained from the Centre for Research on Filariasis and other tropical diseases (CRFILMT), Yaounde, Cameroon. In summary, all the different sera types used have been extensively characterized in a related study [23]. Serum samples for subjects infected with other nematode infections including *Brugia malayi*, *Wuchereria bancrofti*, *Ascaris lumbricoides* and *Mansonella perstans* were obtained from the filarial repository, courtesy of the laboratory of Dr. Steven Williams.

### Sequence analysis and phylogenetic analysis

The amino acid sequence analysis of OvGM2AP was done using Protparam [48] and the sequence alignment of OvGM2AP and homologous sequences from selected nematode species was done using PROMALS3D [49]. Structural prediction analysis was done using the Phyre2 online tool [50]. The phylogenetic tree of OvGM2AP sequences was generated by neighbor joining using PHYLIP 3.695.

### Cloning, expression and purification of OvGM2AP

The full-length OvGM2AP cDNA (WormBase ID: OVOC1952) fused to a C-terminal 8x-His tag was cloned into pAC8_MF, a modified version of the pAC8_H transfer vector [51]. The resulting plasmid (pAC8_MF_OvGM2AP) was co-transfected in insect cells with AcMNPV viral DNA (Bac10:KO_1629,_Δv-cath/chiA-LoxP:DsRed) linearized by Bsu36I to generate the recombinant baculovirus which was subsequently amplified and used to infect large scale cultures for protein production following standardized procedures [52]. Briefly, 1 μg of pAC8_MF_OvGM2AP mixed with 2 μg of linearized viral DNA in 750 μl of the buffer (25 mM HEPES, 140 mM NaCl, 125 mM CaCl_2_, pH 7.1) was added dropwise to 750 μl of Grace’s medium supplemented with 10% of fetal bovine serum (FBS) to obtain a calcium-phosphate precipitate which was allowed to form during 15 min. The precipitate was layered onto 2 x 10^6^ Sf9 cells (Novagen) grown in Grace’s Medium (G8142-SIGMA) previously seeded in a 25 cm^2^ flask. After 4 h incubation at 27°C, the medium was changed and cells further incubated for 5 days at 27°C. The resulting culture supernatant constitutes the initial virus stock (V_0_), which was used to obtain the first (V_1_) and second (V_2_) amplification.

For large scale OvGM2AP production, Sf21 insect cells (IGBMC, Strasbourg) cultivated in the serum free Sf-900 II (Gibco) medium were infected at a density of 1.0x10^6^ cells/ml and MOI of 5 with the V_2_ stock. After 3 days of infection, cells were pelleted by centrifugation (1000 x g for 10 min) and the culture medium was incubated with Ni Sepharose 6Fast Flow (GE Healthcare) at 4 ^o^C for 2 h using 1 mL of resin for 1 L of culture. Following three washes using a 35 mM Imidazole solution in 10% glycerol, 20 mM HEPES pH 7.0, 300 mM NaCl, 5 mM 2-mercaptoethanol (Buffer A) and, bound proteins were eluted with the same buffer in the presence of 500 mM Imidazole. Eluted proteins were dialyzed against Buffer A and snap frozen for storage at -80°C.

Purification of OvGM2AP_8His contained in the clarified lysate was also tested. Cell pellets were re-suspended in a buffer containing 5 mM Imidazole, 10% glycerol, 20 mM HEPES pH 7.0, 300 mM NaCl, 5 mM 2-mercaptoethanol and cells were lysed by sonication at 4°C, 40% Amplitude for 10 sec. The lysate was centrifuged at 20000 g for 30 min and incubated with Ni Sepharose 6Fast Flow (GE Healthcare).

Western blot analysis of the poly-histidine tag from recombinant OvGM2AP_8His was performed using the mouse anti-His antibody 1D11, (IGBMC antibody facility, Strasbourg, France) for primary detection, the donkey F(ab')2 anti-mouse IgG (H+L) conjugated with Horse Raddish Peroxidase (HRP) as secondary antibody (Interchim SA) and the Super Signal West Pico Chemiluminescent substrate for detection of HRP (Thermo Fisher scientific). Chemiluminescence was detected using the Amersham imager 600 QC (GE Healthcare, Sweden).

### Enzymatic deglycosylation of OvGM2AP and mass spectrometry

For glycosylation analysis, purified OvGM2AP was treated with Peptide:N-glycosidase F (PNGase F) (Promega, USA) using a protein/glycosidase ratio of 19/1 (w/w) for 2 h at 37°C. Deglycosylated and undigested OvGM2AP proteins were resolved by SDS PAGE followed by either Coomassie staining or western blot to analyze a band displacement. For mass spectrometry analysis, the proteins were digested by trypsin (after reduction with 10 mM DTT for 1 h at 57°C and alkylation for 45 min in the dark with 55 mM iodoacetamide). Tryptic digests were analyzed using an Ultimate 3000 nano-RSLC (Thermo Scientific, San Jose, California) coupled with a linear trap Quadrupole (LTQ)-Orbitrap ELITE mass spectrometer via a nano-electrospray ionization source (Thermo Scientific). Peptide mixtures were loaded on a C18 Acclaim PepMap100 trap-column (75 μm IDx 2cm, 3μm, 100 Å, Thermo Fisher Scientific) equilibrated with 3% acetonitrile and 0.1% formic acid in H_2_O, and then separated on a C18 Accucore nano-column (75 mm internal diameter (ID)x50 cm, 2.6 mm, 150 Å, Thermo Fisher Scientific) with a 120 min linear gradient from 3 to 80% acetonitrile in the same buffer. Peptides were analyzed by Top 20-CID (collision induced dissociation) data-dependent MS. Spectra were processed with Proteome Discover 2.1 against a protein sequence database for Sf21 cells which include recombinant OvGM2AP. The minimum peptide length required was set to six residues and a minimum of two peptides were required to consider a protein as identified. The protein identification list was filtered at a False Discovery Rate below 1%.

### Peptide synthesis and generation of Polyclonal antibodies (GenScript, USA)

A synthetic peptide was generated following bioinformatics analysis of the OvGM2AP protein using OptimumAntigen design tool. The best peptide was selected on the basis of low host homology and high Antigenicity/Surface/Hydrophilicity index. The synthetic peptide corresponded to amino acid positions 62–72 of the full-length protein with the following sequence from N to C terminal ‘SSKSDGVKFTAEKS’. This synthetic peptide was conjugated to Keyhole Limpet Hemocyanin (KLH) and used to immunize rabbits (GenScript, USA); after a secondary booster, immune serum was collected from rabbits and the antibodies were further purified from the immune serum by affinity chromatography using the synthetic peptide. Approximately 3.5 mg of antibodies was supplied with a titre greater than 1:64,000, affinity purified from 2 rabbits.

### Stage specific expression of OvGM2AP

Total RNA was extracted from the different *O*. *volvulus* parasite stages using the Ambion RecoverAll Total Nucleic Acid Isolation kit (Thermo Fisher Scientific, Belgium). The RNA was reverse-transcribed to cDNA using the iScript cDNA synthesis kit (BioRad, USA). All primers were purchased from Integrated DNA Technologies (IDT, Belgium). Primers were designed targeting *OvGM2AP* cDNA and amplifying a 165 bp product (Forward primer 5’ GCCGAACAGCTCTGGAATTTG 3’, Reverse primer 5’ TCGGTGACATGCGATCAGAC 3’) and these primers were used to amplify the *OvGM2AP* cDNA sequence from the parasite total cDNA as well as the genomic DNA. The designed primer set spans exon 5 to exon 6 (S1 Fig). *O*. *volvulus* Glutaraldehyde-3-Phosphate Dehydrogenase (*OvGAPDH*) was used as a reference gene and a 192 bp fragment was also amplified from cDNA and genomic DNA preparations using the following primers; Forward primer 5’ GAAGGGTGGCGCTAAGAAAG 3’; Reverse primer 5’ GTTGTTGCATGTACGGTGGT 3’. Amplification was carried out using the SensoQuest thermocycler (Göttingen, Germany) under the following conditions: 94 ^o^C for 2 min, 1 cycle; 94 ^o^C for 10 s, 55 ^o^C for 10 s and 72 ^o^C for 1 min, 40 cycles; 72 ^o^C for 10 min. The reaction mix was prepared using the ReadyMix REDTaq PCR reaction mix (Sigma) according to manufacturer’s protocol. The PCR products were run on a 1% agarose gel stained with SYBR safe and visualized under UV light using the Gel Doc XR+ (BioRad, USA).

A western blot analysis of ESPs of different parasite stages was performed using anti-OvGM2AP peptide antibodies (GenScript, USA). Approximately 10 μg of proteins were loaded onto SDS-PAGE gels and run at 200 V. The proteins were transferred to nitrocellulose membranes and blocked with 10% skimmed milk (Régilait, France) overnight at 4 ^o^C followed by incubation with primary antibody at a dilution of 1:2000 for 1 h. After three changes of washing buffer (PBS + 0.05% Tween 20) at 5 min interval each, goat anti-rabbit IgG (whole molecule) HRP conjugate (Sigma) was used as secondary antibody and incubated at a dilution of 1:5000 for 45 min at 37 ^o^C. The membranes were again washed thrice with wash buffer, revealed by chemiluminescence using the ECL substrate and visualized using a C-Digit chemiluminescence scanner (LI-COR, USA).

### Serological characterization of OvGM2AP

IgG response to OvGM2AP was investigated by western blot and indirect ELISA using patient and control sera. For ELISA, optimal antigen concentration was determined by the checkerboard titration method. Maxisorp 96 well microtiter plates (Nunc, Denmark) were coated with 2 μg/ml of purified OvGM2AP overnight at 4 ^o^C. Plates were washed thrice with wash buffer, 5 min between each wash and blocked with 10 mg/ml of Bovine Serum Albumin (BSA) for 1 h 30 min at 37 ^o^C. The plates were washed as above and incubated with the various serum samples as primary antibody at a dilution of 1:2000 for 1 h at 37 ^o^C. Following three rounds of washes at 5 min interval each, the plates were incubated with goat anti-human IgG (Fc Specific) peroxidase conjugate (Sigma) as secondary antibody at a dilution of 1:5000 for 1 h at 37 ^o^C. After a final wash, 3,3’,5,5’ tetramethylbenzidine (TMB) was added as substrate for 10 min at 37 ^o^C. The reactions were stopped with 2M sulfuric acid after which Optical Densities (OD) were read at 450 nm using the iMark microplate reader (BIORAD, USA). All washes and antibody dilutions were done in wash buffer (PBS+0.05%Tween-20).

IgG subclass responses were analyzed as described above with the exception of mouse anti-human IgG subclasses (Sigma) as secondary antibody and an additional incubation step of rabbit anti-mouse IgG peroxidase conjugate (Sigma) as tertiary antibody.

For western blot, approximately 700 ng of purified OvGM2AP was transferred from SDS-PAGE gels to nitrocellulose membranes. The rest of the experimental procedures were carried as described for ELISA above except for the use of 10% skimmed milk (Régilait, France) for blocking and antibody dilution, the use of the Enhanced chemiluminescent (ECL) substrate and revelation using the chemiluminescence scanner.

### GM2AP activity assay

For the assessment of GM2AP activity of OvGM2AP, preparation of human glycosylated recombinant GM2AP with hexahistidine-tag was done as previously described [53]. The enzyme Hex A was purified from human placenta as described for the purification of sphingomyelinase [54]. [^14^C] GM2 was synthesized from its corresponding lyso-lipid, following published procedures [55]. This radiolabeled [^14^C] GM2 was incorporated into large unilamellar vesicles as described before [53,56]. Liposomes contained 20 mol% bis(monoacylglycero)phosphate (BMP) (Sigma, Germany), 5 mol% cholesterol (Sigma, Germany), 1 mol% [^14^C] GM2 and 1,2-Dioleoyl-sn-glycerol-3-phosphocholine (DOPC) (Avanti polar lipids, Alabaster, USA) as a host lipid in 20 mM sodium citrate buffer, pH 4.2. Total lipid concentration was measured to be 50 mM.

The actual liposomal activity assay of GM2AP with Hex A was carried out as previously described [53]. Briefly, 40 μl of liposome dispersion was mixed with 6 mU β hexosaminidase A and for BMP containing vesicles with 4 μg OvGM2AP or 4 μg recombinant human glycosylated GM2AP with a hexahistidine tag made up to 80 μl with 20 mM sodium citrate buffer pH 4.2. The samples were incubated at 37°C for 30 min. Afterwards, the assay was put on ice and stopped by the addition of 20 μl chloroform/ methanol (1/1, v/v). Quantification of the generated [^14^C] GM3 from [^14^C] GM2 was done by thin layer chromatography. The preparations were dried under a stream of nitrogen, re-dissolved with 20 μl chloroform/methanol (1/1, v/v), vortexed and sonified for 15 min after which the solution of lipids was applied to a high-performance thin-layer chromatography plate (Merck, Darmstadt, Germany). Lipids were separated in chloroform/methanol/0.22% CaCl_2_ (55/45/10, v/v/v). Radioactive bands were visualized with a Bio Imaging Analyzer 1000 (Fuji, Japan), and the quantification was performed with the image analysis software ‘‘Tina” (Raytest, Staubenhardt, Germany).

### MUG and MUGS competitive inhibition assay

To assess if OvGM2AP has functional domains analogous to the human orthologue, we employed the use of the artificial fluorogenic substrate 4-methylumbelliferyl-2-acetamido-2-deoxy-β-D-glucopyranoside (MUG) (Sigma, Germany) and its sulfated derivative 4-methylumbelliferyl-2-acetamido-2-deoxy-6-sulfo-β-D-glucopyranoside (MUGS) (Sigma, Germany) in competitive inhibition with OvGM2AP. The assays were performed as previously described [57]. Briefly, a 40 μl reaction mix was constituted for each assay set comprising of 10 mM citrate buffer, pH 4.2, 2 mM MUG or 2 x 10^−5^ M MUGS, 0.250 μg human β-hexosaminidase A (R&D systems, USA), 6 μg BSA (Sigma, Germany) and varying amounts of OvGM2AP. The reaction was incubated for 30 min at 37 ^o^C after which the fluorescence of the released 4-umbelliferone was determined following excitation and emission at 320 nm and 430 nm respectively using a Tecan scanner (Infinite F200 Pro, Austria).

### Statistical analyses

Normality of distributions was assessed using a Shapiro-Wilk test. Normally distributed data are expressed as mean +/- standard deviation and were compared using parametric tests. For non-Gaussian distributions, data are expressed as median with interquartile ranges and were compared using non-parametric tests. Comparisons of more than two groups were made using a one-way analysis of variance (ANOVA) or a Kruskal-Wallis test (with Dunn’s or Tukey’s correction for multiple comparisons) for independent groups as appropriate. The discriminatory performance of total IgG, IgG1, IgG2, IgG3 and IgG4 was assessed using receiver operating curve (ROC) analyses. Areas under the ROC curves (AUCs) were evaluated using the trapezoid method. Standard errors of AUCs were calculated as previously described [58]. Exact confidence intervals for the AUCs were determined using a binomial approach. A p-value < 0.05 was considered statistically significant. Calculations were performed using SigmaPlot for Windows, version 12.5 (Systat Software Inc., Chicago, IL, USA). Diagnostic sensitivity and specificity as well as other diagnostic accuracy parameters were calculated as previously described [59]. Scatter plots were generated using Graph Pad Prism 5.0 (La Jolla, CA, USA).

### Accession numbers

**Table:** 

**No**	**Organism**	**Accession number**	**Database**
1	*Onchocerca volvulus*	OVOC1952	WormBase Parasite
2	*O*. *ochengi*	nOo.2.0.1.g11554	WormBase Parasite
3	*Trichinella pseudospiralis*	T4C_8078.1	WormBase Parasite
4	*Loa loa*	EN70_10408	WormBase Parasite
5	*Caenorhabditis elegans*	C34E7.4	WormBase
6	*Ascaris suum*	ASU_07674	WormBase Parasite
7	*A*. *lumbricoides*	ALUE_0002125701	WormBase Parasite
8	*Toxocara canis*	A0A183UTW5	WormBase Parasite
9	*Dirofilaria immitis*	nDi.2.2.2.g07380	WormBase Parasite
10	*Brugia malayi*	Bm2577	WormBase Parasite
11	*Wuchereria bancrofti*	J9F8Z0	Uniprot
12	*Homo sapiens*	P17900	Uniprot

## Results

### Structural features of OvGM2AP, a putative *Onchocerca volvulus* GM2AP activator protein

**Fig 1:**
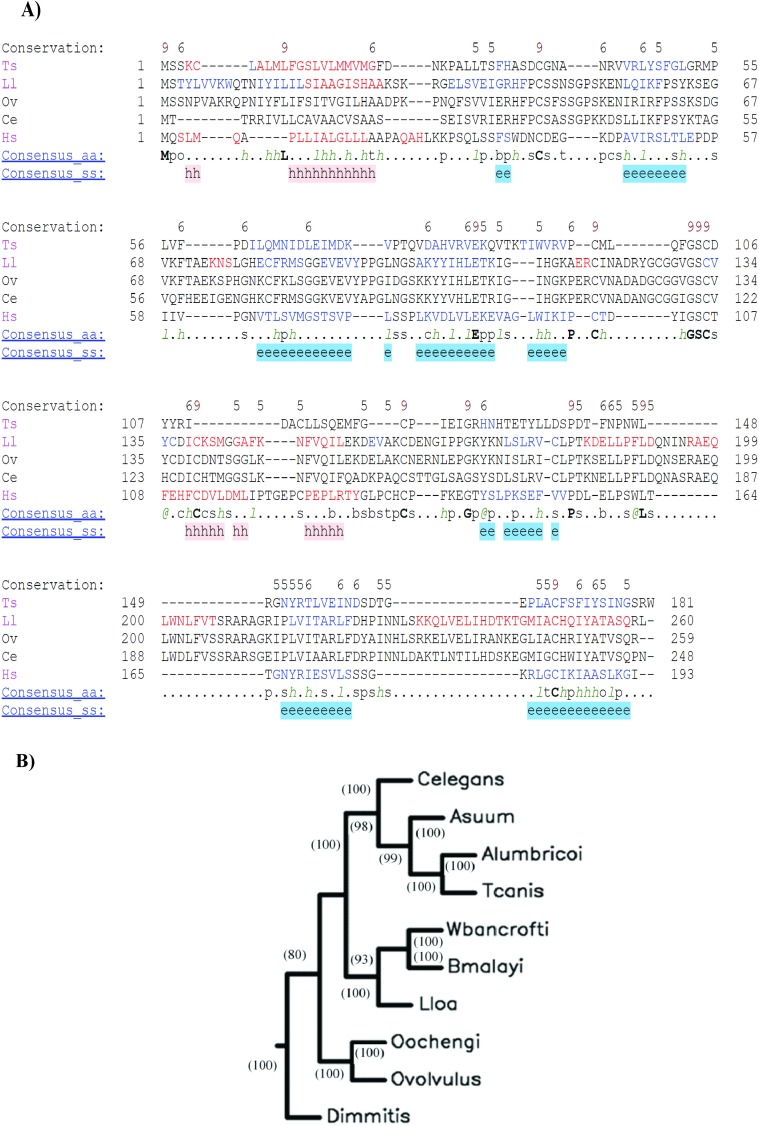
Sequence analysis of OvGM2AP. OvGM2AP protein sequences of the various nematodes were retrieved from WormBase and aligned using PROMALS3D **(A)**. Ov = *Onchocerca volvulus (OVOC1952)*, Ts = *Trichinella pseudospiralis (T4C_8078*.*1)* **16%**, Ll = *Loa loa (EN70_10408)* **72%**, Ce = *Caenorhabditis elegans (C34E7*.*4)* **65%**. Sequence colour representation; red = predicted alpha helices, blue = predicted beta strands; Consensus Structure (ss) symbols: e = beta strand, h = alpha helix. Consensus amino acid symbols: conserved amino acids = bold and uppercase letters; aliphatic (I, V, L): *l*; aromatic (Y, H, W, F): *@*; hydrophobic (W, F, Y, M, L, I, V, A, C, T, H): *h*; alcohol (S, T): o; polar residues (D, E, H, K, N, Q, R, S, T): p; tiny (A, G, C, S): t; small (A, G, C, S, V, N, D, T, P): s; bulky residues (E, F, I, K, L, M, Q, R, W, Y): b; positively charged (K, R, H): **+**; negatively charged (D, E): **-**; charged (D, E, K, R, H): c. Bold residues in the consensus sequence represent greater than 80% consensus. Numbers in the first row represent a level of conservation above 4. Schematic representation of the structure features of OvGM2AP. Phylogenetic analysis of OvGM2AP orthologues **(B)** of closely related nematodes: *Onchocerca volvulus (OVOC1952)*, *O*. *ochengi (nOo*.*2*.*0*.*1*.*g11554)* **99%**, *Dirofilaria immitis (nDi*.*2*.*2*.*2*.*g07380)* **78%**, *Brugia malayi (Bm2577)* **72%,** *Wuchereria bancrofti (J9F8Z0)* **73%**, *Loa loa (EN70_10408)* **72%**, *Ascaris suum (ASU_07674)* **66%**, *Toxocara canis (TCNE_0001193501)* **69%**, *Caenorhabditis elegans (C34E7*.*4)* **65%** *and A*. *lumbricoides (ALUE_0002125701)* **65%**. The Jones-Taylor-Thornton model was used to estimate protein distances for neighbor joining. Bootstrap values are indicated on the nodes.

**Fig 2:**
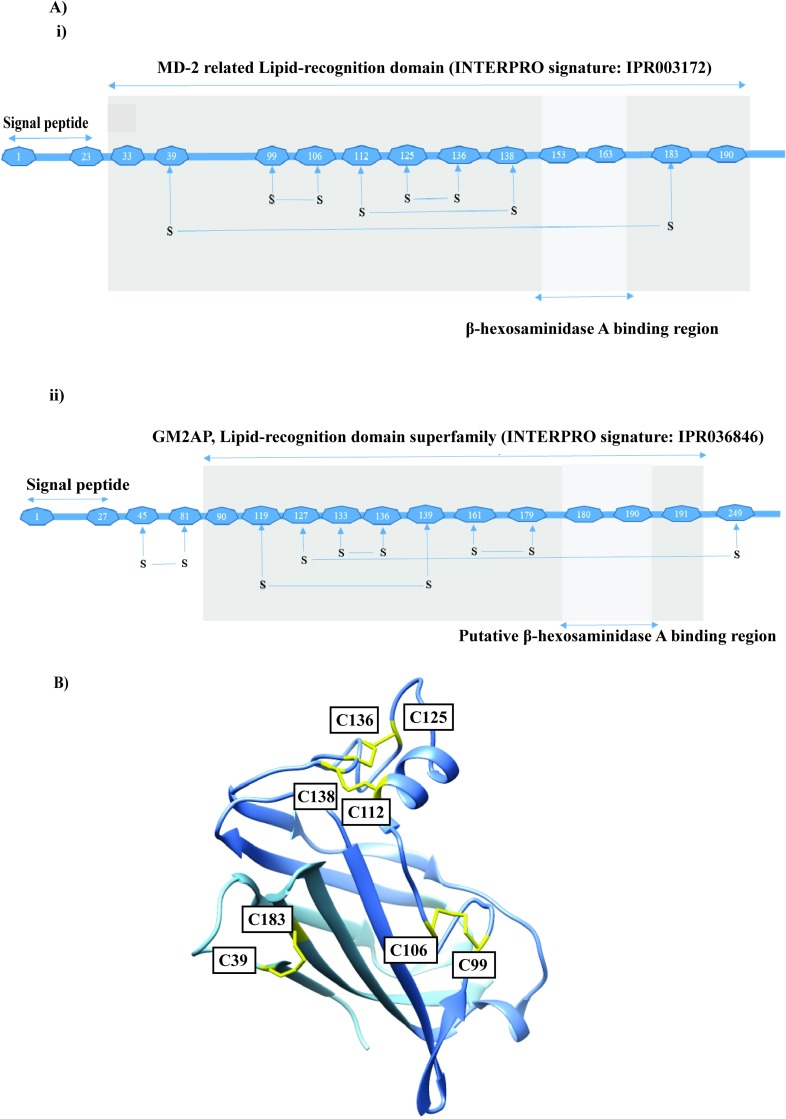
Structural annotations of the GM2AP. Representation of human GM2AP domain organization (**A(i)**). Predicted lipid binding domain and disulfide bond positions for OvGM2AP are indicated as well (A(ii)). Disulphide bonds were predicted using the DISULFIND online prediction tool. 3D structure of human GM2AP (PDB access code 1G13) **(B)**.

The complete cDNA sequence coding for OvGM2AP was retrieved from NCBI non-redundant database by blasting the reverse complementary of the partial sequence referred to as OvL3.C1 [60]. The full-length protein is assigned a WormBase ID of OVOC1952. It contains 259 amino acids comprising 10 cysteine residues, 6 of which occupy the same positions in other species such as *Homo sapiens*, *Loa loa*, *Trichinella pseudospiralis*, and the free-living *Caenorhabditis elegans* (Fig 1A). INTERPRO analysis of this protein revealed that it belongs to the superfamily of the GM2 activator protein and has a lipid binding domain. SUPERFAMILY, a member database of InterPro which is a library of profile hidden Markov models representing all proteins of known structure, was used to construct the entry. This same analysis holds true for orthologues of the protein in other nematode species and remains uncharacterized in all these species, except for *T*. *pseudospiralis*. Gene Ontology suggest it may be involved in biological processes such as nematode larval development, body morphogenesis, molting cycle, growth and locomotion. The degree of relatedness of OvGM2AP with orthologues in other nematodes depicts a conservation of the protein across species and further supports *O*. *ochengi* as the closest relative of *O*. *volvulus* (Fig 1B). The protein sequence was found to contain a signal peptide (amino acids 1–27 for OvGM2AP and 1–23 for human GM2AP) in most orthologues. The amino acid sequences corresponding to the β-hexosaminidase A binding domain for the human GM2AP as well as the putative β-hexosaminidase A binding domain for OvGM2AP are illustrated in Fig 2A. Phyre2, a structure prediction online tool not affiliated to the INTERPRO consortium, equally predicted OvGM2AP as a GM2AP with greater than 90% confidence. The complete list of hits obtained from the Phyre2 blast have been compiled in S2 Table. A model of the structure of the human GM2AP template is indicated in Fig 2B

In terms of amino acid composition of the protein, serine was the most abundant, with 23 residues (8.9%). Other amino acids with structural implications present in the protein include Glycine (7.3%) and Proline (5.8%).

### Transcriptional and translational expression patterns of OvGM2AP gene

**Fig 3:**
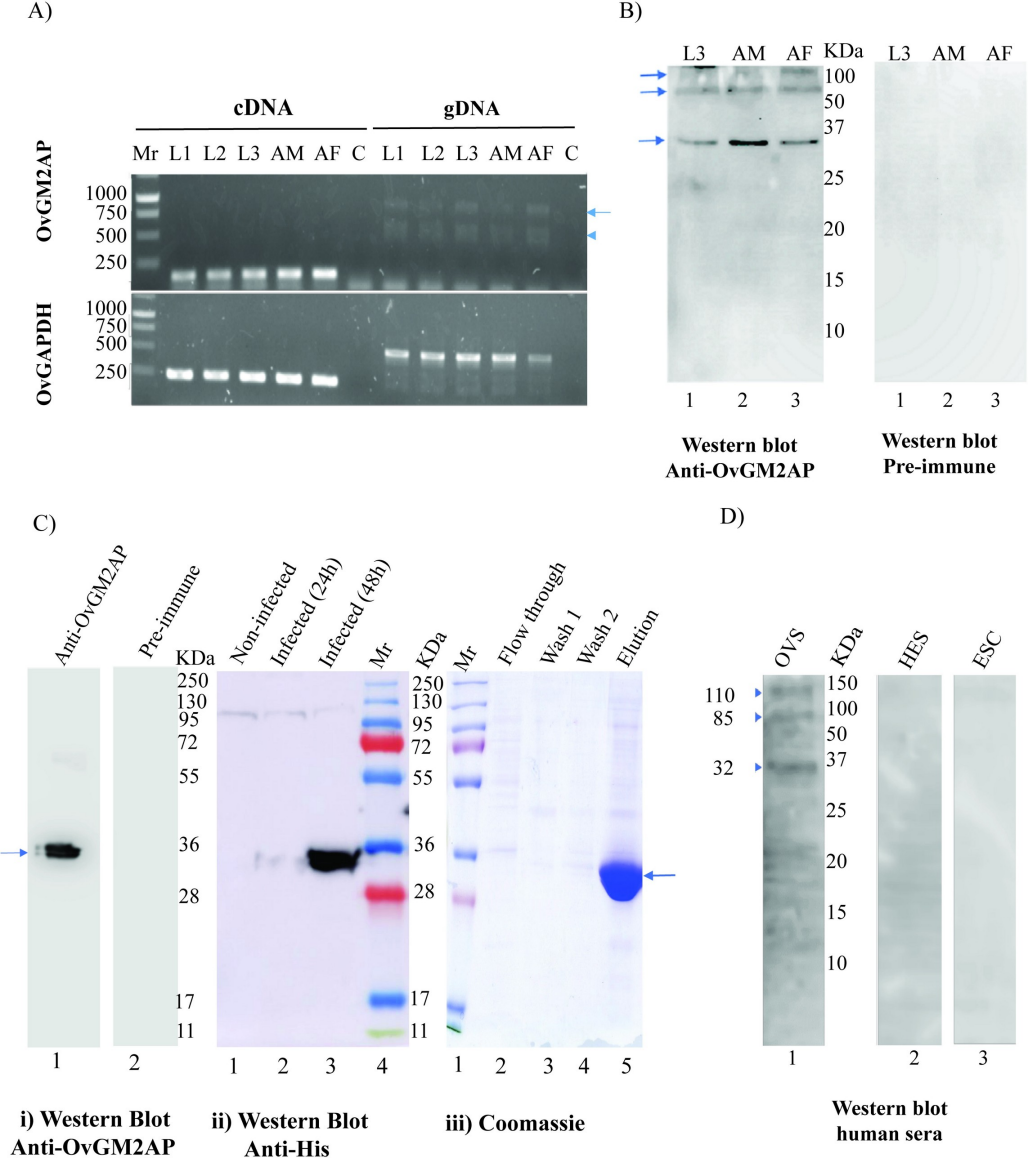
Expression profile of *OvGM2AP*. Transcriptional expression of OvGM2AP and OvGAPDH was evaluated from L1, L2, L3, adult male (AM) and adult female (AF) parasite stages by RT-PCR **(A)**. PCR products were run on 1% agarose gels. Primers were designed for the specific amplification of 165 bp sequences of *OvGM2AP* and 192 bp sequences of *OvGAPDH*. *OvGAPDH* was used as a normalization control. OvGM2AP was also amplified from genomic DNA (gDNA) of the various parasite stages as control. Arrow indicates 957 bp target amplicon from gDNA while arrow head indicates a possible non-specific PCR product of approximately 600 bp. Mr = Promega 1Kb DNA ladder, C = No Template Control. OvGM2AP was also detected in 16 h ESP by western blot analysis using anti-OvGM2AP peptide antibody as primary antibody **(B)**. Arrows indicate signals of OvGM2AP indicating the 28.8 KDa protein and possible oligomeric forms at approximately 85 and 110 KDa. The recombinant OvGM2AP expressed in Sf21 cells was analyzed by western blot after purification from IMAC Ni-NTA resin with 500 mM Imidazole **(C).** The flow-through (FT), the two wash fractions (W1 and W2) and the eluate (E) were analyzed by electrophoresis on a coomassie blue stained polyacrylamide gel (**iii**). Recombinant OvGM2AP was detected using serum from a rabbit immunized against an OvGM2AP peptide (anti-OvGM2AP) or a rabbit pre-immune serum (PI) (**i**). Sf21 cells, un-infected (lane 1) or infected by the recombinant virus expressing OvGM2AP were harvested 24 or 48 hours (lanes 2 and 3) after infection. The corresponding lysates were analysed by western blot using a mouse anti-His monoclonal antibody (1D11) (**ii**). Total IgG responses to the purified recombinant protein was analysed **(D)** using the indicated serum types (ESC = European serum control, HES = Hypoendemic serum and OVS = *O*. *volvulus* serum) as primary antibodies by western blot. Arrow heads indicate positions of OvGM2AP around 37 kDa and possible oligomers of around 85 and 110 kDa. A 12% SDS-PAGE gel was prepared in every instance of gel preparation using either a 40% acrylamide stock (C) or a 30% acrylamide stock (B and D).

In order to determine the timing of OvGM2AP transcriptional expression during the various life cycle stages of the parasite, total RNA was purified from the larval stage 1 (L1) or Mf, stage 2 (L2), stage 3 (L3), adult male (AM) as well as adult female (AF) stages and analyzed by RT-PCR using primers targeting OvGM2AP exon 5 (forward primer) and exon 6 (reverse primer) and generating a 165 bp product. Results obtained reveal that OvGM2AP mRNA is present in L1, L2, L3, adult male (AM) and adult female (AF) stages of *O*. *volvulus* (Fig 3A). To confirm that the detected bands of interest were not resulting from DNA contamination of the samples, the same primer pairs were also used to analyze *O*. *volvulus* genomic DNA (gDNA). As shown in Fig 3A (right panel), our primers detected signals with higher sizes, consistent with the genomic position of the targeted exons separated by intron 5 (S1 Fig). The size difference between RNA and gDNA samples was also visible using *O*. *volvulus* GAPDH primers as control (Fig 3A, lower panels), again consistent with the genomic positions of the targeted exons. The primer set designed to amplify OvGM2AP in *O*. *volvulus* samples was also successfully used to amplify the gene in *O*. *ochengi* species with exact precision as in *O*. *volvulus* further supporting the genetic closeness between the two species. In conclusion, the presence of the transcript in all the analyzed parasite stages suggests its permanent expression, which required confirmation at the translation level. In order to address this translational expression experimentally and since *in silico* analysis of OvGM2AP protein sequence suggests that it is a secreted protein, L3, AM and AF *O*. *volvulus* worms were cultured *in-vitro* for 16 h and their corresponding ESPs were analyzed by western blotting using an anti-OvGM2AP peptide antibody. OvGM2AP was detected in 16 h *in-vitro* ESPs of third stage larvae (L3), AM and AF with the anti-OvGM2AP peptide antibodies but not with the pre-immune serum (Fig 3B). This suggest that OvGM2AP is expressed and secreted by the parasite in its host at the tested life cycle stages and likely at all the different stages as revealed by RT-PCR analysis above.

### Mass expression and purification of recombinant OvGM2AP from insect cells

A constant and reliable source of recombinant antigen is required for in depth analysis of the OvGM2AP antigen. Attempts to produce soluble recombinant OvGM2AP in *E*. *coli* were only partially successful as mass spectrometry analysis of the purified protein showed the dominance of chaperones (S2 Fig), we therefore switched to the insect cell baculovirus expression system. A recombinant virus encoding the OvGM2AP in fusion with a C-terminal 8His-tag under the control of the late pH promoter was generated by homologous recombination and was tested for its capacity to produce soluble OvGM2AP. This led to the production of the full-length protein including the TEV cleavage site and 8x His expected with a MW of 32 KDa (Fig 3C(iii)). Western blot analysis of the clarified lysate from cells infected by the recombinant virus detected a protein of approximately 35 kDa that reacted with the serum of a rabbit immunized against an OvGM2AP peptide but not with the pre-immune serum (Fig 3C(i)).

As OvGM2AP is a secreted protein with a putative classical signal peptide, IMAC affinity purification of the histidine-tagged recombinant protein was assessed from cell lysates as well as from the culture medium. The latter purification strategy was successful and purification of OvGM2AP_8His from the culture medium using a single Ni-NTA affinity step yielded a pure protein (Fig 3C) with a concentration close to 1 mg per liter of culture. Mass spectrometry confirmed the identity of the purified protein with 23 unique peptides detected and a sequence coverage of 78.88% (S3 Table). We could not detect any peptide corresponding to the N-terminal signal sequence of OvGM2AP as identified by sequence analysis. This suggests the peptide was cleaved and could constitute the signal peptide sequence of the protein.

We next investigated the antigenic potential of the insect cell expressed and purified recombinant OvGM2AP. We first asked if the protein, new for analysis in *Onchocerca*, could distinguish between onchocerciasis patients and onchocerciasis non-infected individuals. When a 14 sera pool of *O*. *volvulus* Serum (OVS) and Hypoendemic Serum (HES) control, as well as a 3 sera pool of European serum control (ESC) were employed for western blot analysis of 700 ng of purified OvGM2AP, OvGM2AP was found to react specifically with patient sera (OVS) and not with HES and ESC controls (Fig 3D). The presence of other bands of higher molecular weight suggests a possible polymerization of the native protein, as also observed with the *in vitro* ESP analysis above (Fig 3B).

### The asparagine at position 173 of the recombinant OvGM2AP is glycosylated

**Fig 4:**
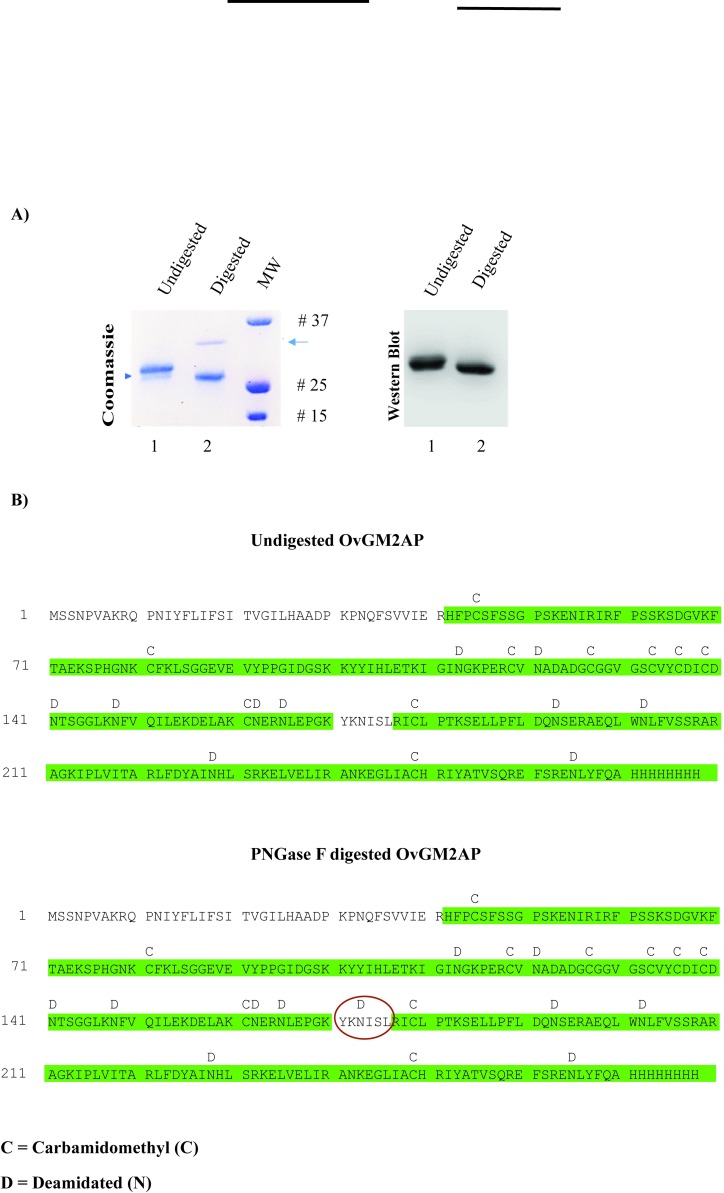
PNGase F Digestion analysis of OvGM2AP. OvGM2AP was subjected to PNGase F digestion and analyzed on Coomassie blue stained SDS-PAGE gel and on nitrocellulose membranes by western blot **(A)**. Arrow represents PNGase on coomassie staining which could not be detected using anti-OvGM2AP peptide antibodies on western blot. Protein exist predominantly in glycosylated form in the undigested fraction with a relatively small part of it unglycosylated (arrow head). MS analysis of the corresponding peptides revealed the presence of a deamidation at asparagine 173 in the deglycosylated (digested) protein but not in the glycosylated (undigested) protein **(B)**; red circles indicate point of deamidation.

The *Homo sapiens* GM2AP is known to be glycosylated at asparagine 63 [61]. There is experimental evidence of glycosylation of the *C*. *elegans* orthologous protein at asparagine 85 [62]. It therefore appears that the glycosylated asparagine amino acid residue differs across species and also between different members of the family. We herein analyzed the recombinant OvGM2AP produced in insect cell for the possible presence of glycosylation. To achieve this, Peptide:N-glycosidase F (PNGase F) was used to digest the recombinant OvGM2AP and the digestion product was resolved by SDS-PAGE. As shown (see arrow head) in Fig 4A(i), a band shift was observed between the digested and undigested purified protein. This band shift was confirmed by western blot analysis using the anti-OvGM2AP peptide antibody (Fig 4A(ii)). The band corresponding to the enzyme used in the digestion (see arrow, Fig 4A(i)) failed to show a signal by western blot, further testifying to the specificity of our anti-OvGM2AP peptide antibody.

To verify if the shift was associated with enzymatic deglycosylation, mass spectrometry analysis was performed on peptides obtained from trypsin digestion of PNGase F treated and untreated OvGM2AP samples. The asparagine at position 173 in the native recombinant OvGM2AP was found to be deamidated to aspartate in the digested protein (Fig 4B), a consequence of PNGase F digestion of N-linked glycans from glycoproteins. Two different glycosylation sites were observed by mass spectrometry with 100% and 86% confidence corresponding to the peptide “NISLRICLPTK” and peptide “NLEPGKYKNISLR” respectively. This PNGase F treatment therefore suggests that the purified recombinant OvGM2AP is glycosylated predominantly at Asn 173 but with the possibility of a second glycosylation site at Asn 165. The ion exchange chromatogram corresponding to the deamidation peaks of the identified glycosylated peptides is provided in S3 Fig. Altogether, this supports the glycosylation of the protein at asparagine 173 following the pattern N-X-S where X is any amino acid except proline.

### The humoral immune response to OvGM2AP divulges its immunogenicity

**Fig 5:**
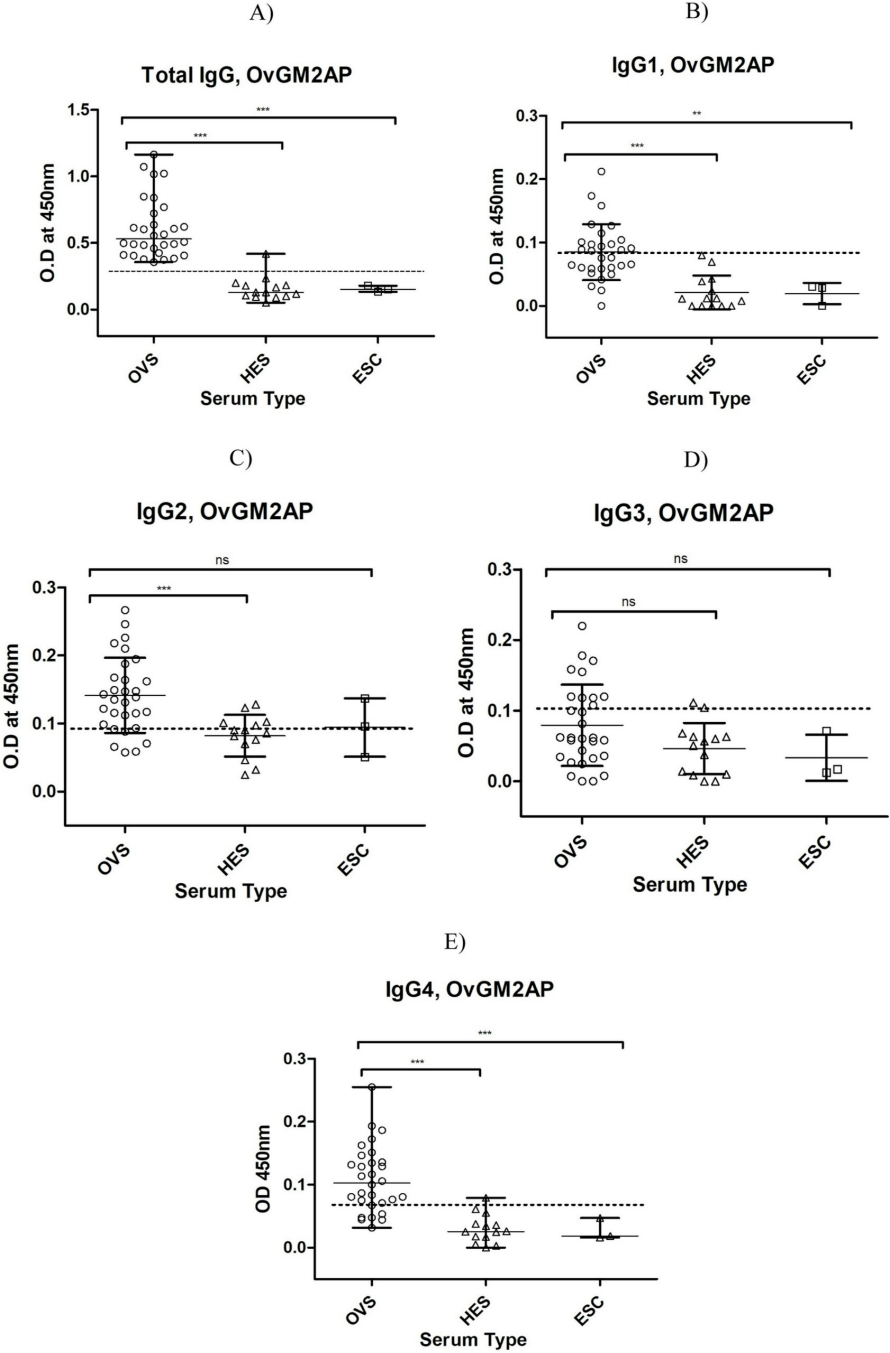
Analysis of humoral immune response to OvGM2AP. Purified 8X His-fused OvGM2AP was used to coat microtiter plates. Plates were blocked and later incubated with serum from indicated individuals (OVS, HES, or ESC), followed by secondary antibody of either goat anti-human IgG peroxidase conjugate **(a)** or mouse anti-human IgG1-4 **(b-e)** which was followed with goat anti-mouse IgG, peroxidase conjugate as tertiary antibody. The plates were revealed using TMB. Optical density of stopped reactions was read at 450 nm and OD values were plotted against the different serum types. OVS = *O*. *volvulus* Serum (n = 30), HES = Hypo-endemic Serum (n = 14), ESC = European serum control (n = 03). A one-way ANOVA was used to compare groups. Error bars represent median with range. Dotted horizontal lines represent cut-off at mean + 3 SD of HES.

As an attempt to understand the immune response to OvGM2AP, total IgG responses were measured by ELISA in infected and non-infected individuals. Results obtained indicate, consistent with the western blot analysis reported above (Fig 3D), a discriminatory immune response to OvGM2AP between infected and non-infected individuals. The mean OD_450nm_ for patient sera (OVS) was significantly different (P<0.05) from that of normal African sera (HES) and European sera (ESC) with p-values lower than 0.001 for both HES and ESC (Fig 5A). The area under the ROC curve (AUC) was found to be high, with a value of 0.9863, and a p-value < 0.0001 (Table 1; S4 Fig) indicating both high sensitivity and specificity.

**Table 1:** Table of ROC values for IgG and IgG subclass responses to OvGM2AP. ROC values were calculated for IgG and IgG subclass. Other parameters such as standard error (SE), confidence interval (CI), and P-values were also calculated for the IgG and IgG subclass immune responses to OvGM2AP. AUC = Area under the ROC curve.

ROC curve analysis	Type of Immune Response				
	Total IgG	IgG1	IgG2	IgG3	IgG4
ROC curve area (AUC)	0.9863	0.9078	0.8118	0.6598	0.9451
SE of AUC	0.01448	0.04605	0.06174	0.08077	0.03008
95% CI of AUC	0.96–1.02	0.82–0.99	0.69–0.93	0.50–0.82	0.89–1.00
P-value (against AUC = 0.5)	< 0.0001	< 0.0001	0.0004	0.07	< 0.0001

We further investigated IgG subclass responses to the recombinant OvGM2AP by ELISA. All IgG subclasses (with the exception of IgG3) were detected at significantly different levels between infected individuals (OVS) and uninfected African Controls (HES) (Fig 5B–5E). The IgG4 subclass was found to have a higher AUC value (0.9451) compared to the other subclasses with a p-value < 0.0001 (Table 1). Diagnostic accuracy parameters investigated for the IgG and IgG subclass responses also revealed IgG4 as the favorite (Table 2).

**Table 2:** Diagnostic accuracy parameters for OvGM2AP. Cut off values were obtained at mean + 3 standard deviations of the hypoendemic serum (HES). Sensitivity refers to the probability of getting a positive test result in subjects with the disease; Specificity is the probability of a negative test result in a subject without the disease; Positive predictive value defines the probability of having the disease of interest in a positive result; Negative predictive value is the probability of not having the disease in a subject with a negative test result; Likelihood ratio for positive test results indicates how much more likely the positive test result is to occur in subjects with the disease compared to those without the disease; Likelihood ratio for negative test results represents the ratio of the probability that a negative test result will occur in subjects with the disease to the probability that the same result will occur in subjects without the disease.

Diagnostic Accuracy Parameter	Type of Immune Response				
	Total IgG	IgG1	IgG2	IgG3	IgG4
Sensitivity (%)	100	50	80	30	76.7
Specificity (%)	92.8	100	64.3	92.8	92.8
Positive Predictive Value	0.96	1	0.83	0.9	0.96
Negative Predictive Value	1	0.48	0.6	0.38	0.65
Likelihood ratio for positive test result	13.89	0	2.24	4.17	10.653
Likelihood ratio for negative test result	0	0.5	0.311	0.754	0.251
Cut off value	0.2712	0.08	0.092	0.109	0.068

### Levels of OvGM2AP antibodies are inversely correlated with duration of ivermectin treatment but cross-reacts with serum samples from *L*. *loa* patients

**Fig 6:**
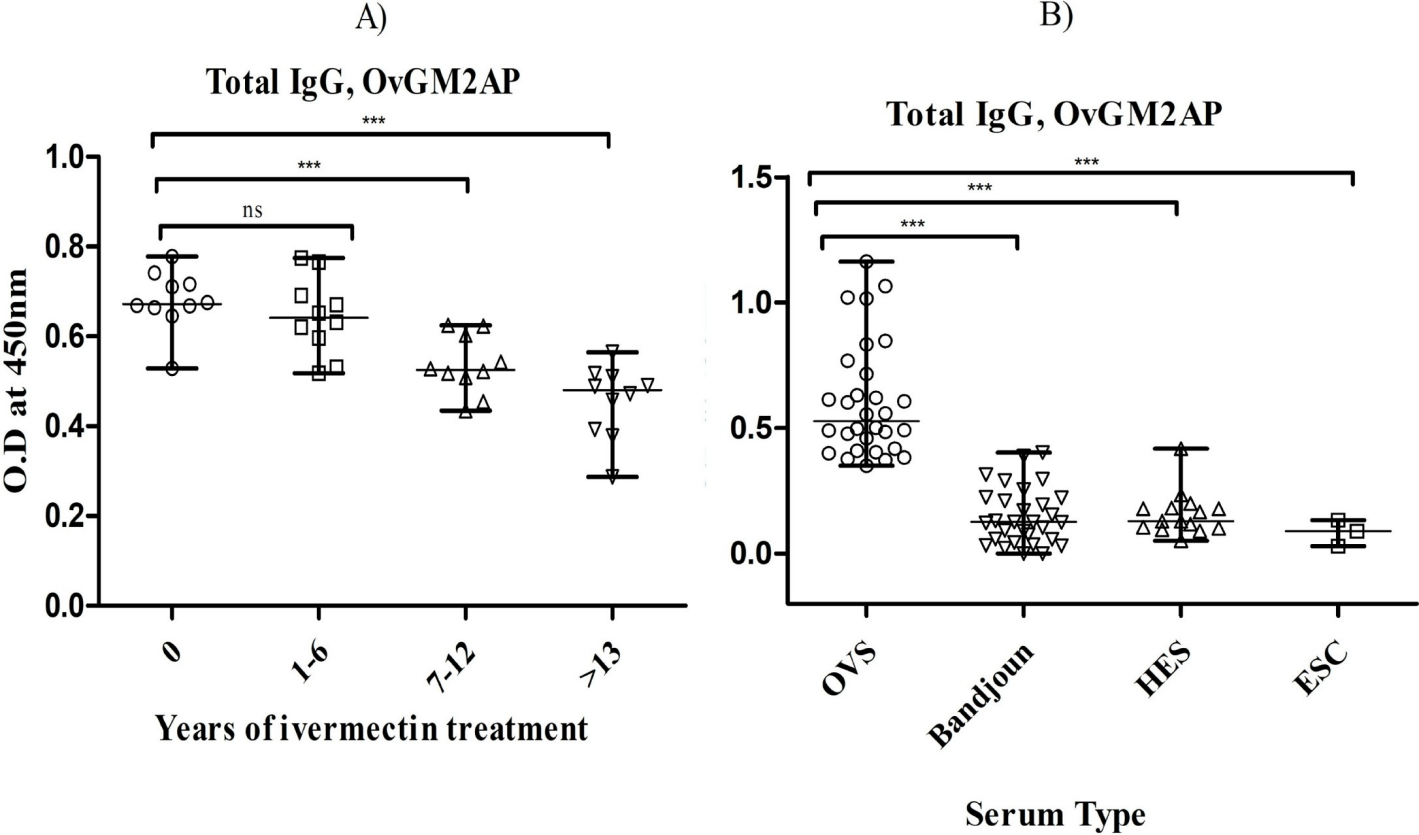
ELISA-analysis of IgG responses of treated individuals to OvGM2AP. Purified 8X His-fused OvGM2AP was used to coat microtiter plates. Plates were blocked and later incubated with the indicated categories of serum followed by secondary antibody (goat anti-human IgG peroxidase conjugate). The plates were revealed using TMB. Optical density of stopped reactions was read at 450 nm and OD values were plotted against ivermectin treatment rounds (a) or different the different serum types (b). a) IgG responses to OvGM2AP in patients (n = 40) with different rounds of ivermectin intake including groups of ivermectin naïve patients, 0 (n = 10), 1–6 (n = 10), 7–12 (n = 10) and >13 (n = 10) rounds of ivermectin administration. b) IgG responses to OvGM2AP in different patient groups. OVS = *O*. *volvulus* Serum (n = 30), Bandjoun = serum samples from Bandjoun (n = 30), an onchocerciasis hypo-endemic zone, HES = Hypoendemic Serum (n = 14), ESC = European serum control (n = 03). A one-way ANOVA was used to compare groups. Error bars represent median with range.

In a bid to assess OvGM2AP as a possible marker of a decrease of infection related to ivermectin treatment, we decided to investigate the correlation between the humoral immune response and rounds of ivermectin (IVM) intake. To this end, sera from Bandjoun in the Western region of Cameroon where onchocerciasis is almost eliminated [46] were grouped (10 samples per group) according to the number of rounds of ivermectin intake (expressed as years of treatment) and analyzed by ELISA using OvGM2AP as a bait. As shown in Fig 6A, results obtained suggest a negative correlation between the anti-OvGM2AP response and rounds of IVM intake. Consistently, samples from this site were compared to those from Kombone in the South-Western region where onchocerciasis remains actively endemic. In the analysis, sera from Rwanda where this disease is hypo-endemic (HES) were used as negative controls together with European sera (ESC). As shown in Fig 6B, OvGM2AP significantly discriminated between sera from the endemic region of Kombone (OVS) and the hypoendemic region of Bandjoun. This suggests that monitoring anti-OvGM2AP immune response would allow the discrimination of endemic and hypoendemic regions as well as the evaluation of the state of infection following ivermectin treatment.

**Fig 7:**
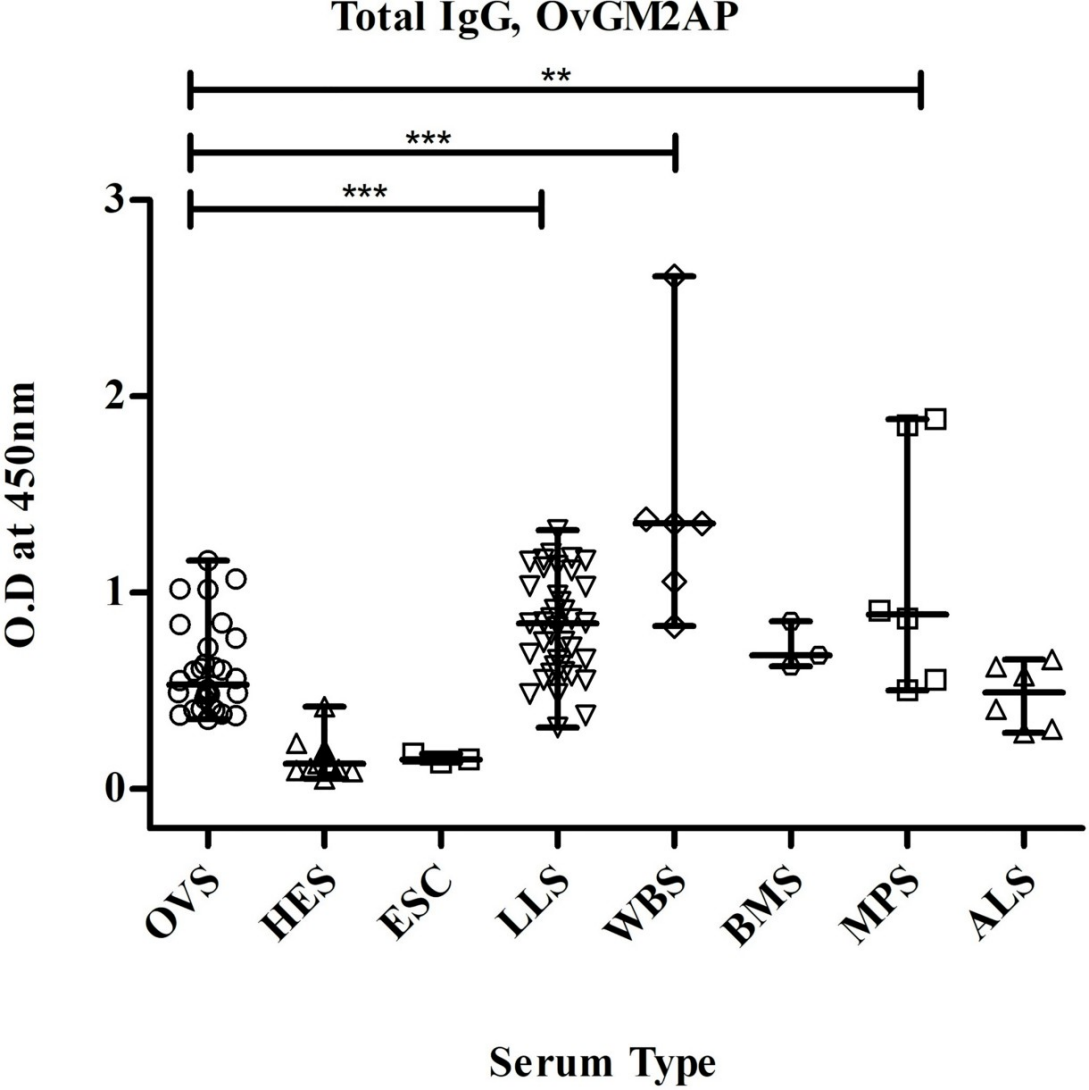
Analysis of humoral immune response of related nematode sera to OvGM2AP. Purified 8X His-fused OvGM2AP was used to coat microtiter plates. Plates were blocked and later incubated with serum from indicated individuals (OVS, HES, LLS, WBS, BMS, MPS, ALS or ESC), followed by incubation with goat anti-human IgG peroxidase conjugate as secondary antibody. The plates were revealed using TMB. Optical density of stopped reactions was read at 450 nm and OD values were plotted against the different serum types. OVS = *O*. *volvulus* Serum (n = 30), HES = Hypo-endemic Serum (n = 14), ESC = European serum control (n = 03), LLS = *L*. *loa* serum (n = 39), WBS = *W*. *bancrofti* serum (n = 06), BMS = *B*. *malayi* serum (n = 03), MPS = *M*. *perstans* serum (n = 06), ALS = *A*. *lumbricoides* serum (n = 06). A one-way ANOVA was used to compare groups. Error bars represent median with range. Dotted horizontal lines represent cut-off at mean + 3 SD of HES.

In order to investigate a possible cross-reaction with OvGM2AP in individuals infected with other nematodes, we analyzed sera from patients infected with *Loa loa*, *Brugia malayi*, *Wuchereria bancrofti*, *Mansonella perstans* and *Ascaris lumbricoides*. With this in mind, OvGM2AP was used to coat ELISA plates as earlier described and IgG response was measured in *L*. *loa* infected serum (LLS), *W*. *bancrofti* infected serum (WBS), *B*. *malayi* infected serum (BMS), *M*. *perstans* infected serum (MPS), *A*. *lumbricoides* infected serum (ALS) and compared with OVS and controls. Results obtained indicated a very strong response from related nematodes (Fig 7). This suggests consistently with the high sequence identity shown above (Fig 1A), that epitopes are common to Ov and Ll GM2AP, as well as the other nematodes. This is a drawback for the use of OvGM2AP in the specific diagnosis of the disease.

### OvGM2AP could not hydrolyze the GM2 to GM3

The ability of OvGM2AP to mediate the hydrolysis of GM2 by β-hexosaminidase A was investigated in an *in vitro* liposomal assay using negatively charged, BMP-containing liposomes. Liposomal composition was Cholesterol (5 mol%), BMP (20 mol%) and [^14^C] GM2 (1 mol%), made up to 100 mol% by DOPC.

**Fig 8:**
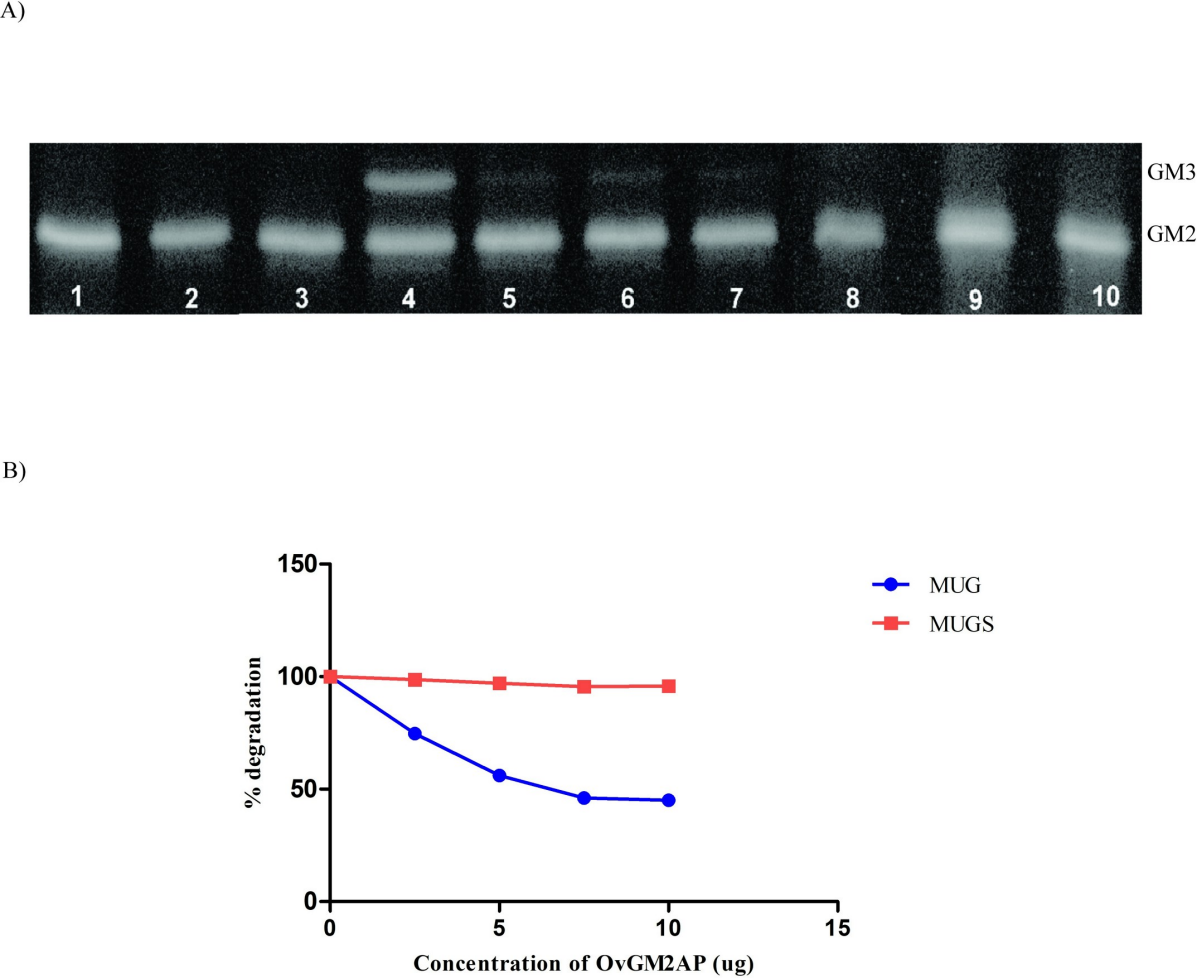
Analysis of GM2AP activity of OvGM2AP. The ability of OvGM2AP to hydrolyze GM2 to GM3 was assessed in a liposomal setting **(A)**. Liposomes were incubated with the following accordingly to the numbers; 1 = human recombinant GM2AP (negative control), 2 = OvGM2AP (negative control), 3 = β-hexosaminidase A (negative control), 4 = β-hexosaminidase A and tauridesoxicolate (positive control), 5–7 = β-hexosaminidase A and human recombinant GM2AP (positive control), 8–10 = β-hexosaminidase A and OvGM2AP. Varying concentrations of OvGM2AP were incubated with recombinant Hex A and MUG or MUGS artificial substrate **(B)** and levels of degradation of the substrates was assessed using a fluorimeter.

Turnover of [^14^C] GM2 in the liposomal activity assay was stimulated by human recombinant GM2AP or tauridesoxicolate (TDC) (50 μg), both used as positive control. No hydrolysis of [^14^C] GM2 was detected in the presence of OvGM2AP (Fig 8A).

### OvGM2AP competitively inhibits MUG degradation by recombinant β-hexosaminidase A but not MUGS

The human GM2AP has been reported to competitively inhibit the degradation of the artificial fluorogenic MUGS substrate but not the neutral MUG substrate. In a bid to understand if a parasitic GM2AP like OvGM2AP could share the same characteristics like the human GM2AP, we employed MUG and MUGS in a competitive inhibition assay with OvGM2AP. As shown in Fig 8B, OvGM2AP was found to competitively inhibit the degradation of MUG, but not MUGS as previously reported for the human GM2AP [63].

## Discussion

Parasite genomics has evolved drastically within the last decade with more genomes being sequenced. The recently published genome of *Onchocerca* revealed a 44% of *O*. *volvulus* genes coding for proteins with no predicted function [21]. In order to deeply understand the biology of the parasite, these unknowns should be solved. In this study, we attempted to characterize one such uncharacterized protein belonging to the *O*. *volvulus* ESP family which was identified by INTERPRO analysis to be a member of GM2 activator proteins (GM2APs).

The canonical GM2APs are accessory glycoproteins required for the *in vivo* degradation of ganglioside GM2 to GM3 in the lysosomal compartments by β-hexosaminidase A [64]. The latter can cleave glycolipid substrates on membrane surfaces only if they extend far enough into the aqueous phase. In the absence of detergents, the degradation of ganglioside GM2 occurs only in the presence of the GM2 activator protein [41]. GM2AP has also been shown to act as a lipid transfer protein [37,38]. Although INTERPRO analysis identified Ov28CRP as a GM2AP, its size is a little bigger and the amino acid identity is very low, suggesting the *O*. *volvulus* protein could be a GM2AP member with novel functions. Moreover, we have provided experimental evidence of its secretion in 16 h *in vitro* ESPs collected from L3, adult male and adult female stages, supporting the presence of the protein in a parasitic ESP *in vivo*. *Trichinella pseudospiralis* GM2AP has been reported to be secreted following its expression in yeast cells. This parasite GM2AP did not, however, facilitate degradation of GM2 ganglioside by N-acetylhexosaminidase A, although it did inhibit phospholipase D (PLD) activity. Lack of the former activity might be explained by the absence of a domain implicated in binding to hexosaminidase [65]. In this study, we also did not observe hydrolysis of GM2 to GM3 by OvGM2AP, supporting a trend that this activity might be lost in parasitic species. Additionally, we observed competitive inhibition of MUG degradation with OvGM2AP (Fig 8B) and not MUGS as previously reported for the human GM2AP [63]. The competitive inhibition of MUGS with the human GM2AP was proposed to be as a result of the binding of GM2AP/MUGS to a site on the α subunit of β-hexosaminidase A (Hex A). However, a recent model of GM2AP/GM2/Hex A was provided, predicting the binding of GM2AP to a site on the β subunit of HexA while it presents the GM2 to a positively charged site on the α-subunit which interacts with the negatively charged sialic acid of GM2 [66]. The inhibition of degradation of MUG which is a neutral substrate by OvGM2AP indicates the possible lack of a positively charged site in OvGM2AP that binds to the negatively charged MUGS substrate. This is a first indication of the protein playing an opposite role to that previously described for the human protein and could therefore be conferring some advantages in supporting a parasitic lifestyle. Another possible function of the GM2AP with potential significance in host-parasite interactions is phospholipase D (PLD) inhibition. PLD is involved in the signaling pathway, hydrolyzing the phosphodiester bond of the glycerolipid phosphatidylcholine to second messenger phosphatidic acid (PA) [67]. PLD and its second messenger PA have been implicated in a variety of biological processes including signal transduction and anti-apoptotic signaling, phagocytosis, exocytosis/secretion, as well as chemotaxis for neutrophils and cancer cells [68–73]. The *E*. *coli* expressed non-glycosylated human GM2AP can be recaptured from the extracellular medium into the cell [74] and this was reported to be mediated mainly through the mannose-6-phosphate pathway [75]. Investigating the ability of OvGM2AP to be internalized by immune cells will throw light into its possible immunomodulatory functions. It was also demonstrated that the binding abilities of the GM2AP are altered by the presence of a His-tag [76]. These observations provide critical information to take into account in our future biochemical characterization of the *O*. *volvulus* His-tagged recombinant OvGM2AP. In addition, and contrary to data on the human GM2AP, the nematode homologue failed to inhibit platelet activating factor-induced calcium mobilization in neutrophils, but actually enhanced mediator-induced chemotaxis [65].

As OvGM2AP is secreted, its presence in key parasite stages can be useful in the development of an antigen capture test as this would make it possible to detect the presence of L3s, adults and microfilariae in infected individuals thereby increasing its scope in detecting both pre-patent and active infection.

While OvGM2AP functional elucidation remains a topic for a separate investigation, we have been able to characterize anti-OvGM2AP humoral immune responses using the recombinant protein expressed in SF21 insect cells. Our choice of insect cells in which posttranslational modifications are possible was dictated by a gain of knowledge of the immune response to a fully functional molecule. Analysis of the total IgG response to OvGM2AP revealed it as discriminant between infected and non-infected individuals. We furthermore analyzed the immune response to OvGM2AP in patients who have been administered different rounds of Ivermectin and found a statistically significant decline in IgG response with increased rounds of drug administration (Fig 6A). This finding is useful in the context of the assessment of onchocerciasis control programs for which skin snip Ov16 based test are still being widely used for evaluation of the CDTI program in the definition of treatment endpoints [77]. Limitations of both tests have called for the search of new diagnostic antigens and thus allowing the design of antigen cocktails that may solve the present difficulties in obtaining high specificity without a corresponding drop in sensitivity [18]. The use of OvGM2AP as a constituent of antigen cocktails to determine ivermectin treatment endpoints might improve on its major drawback which is its cross-reactivity with related nematode infections, limiting its analytical specificity.

Helminth parasites are known to modulate host immune responses to establish their long term stay in an immunocompetent host. Individuals with high parasite load are often asymptomatic and present only mild pathology. This is largely due to the fact that the immune response to helminth infection is less severe and regulatory [78]. Indeed, the immune response is usually T_H_2 biased, with a T_H_2 response shift either towards immunosuppression, immuno-tolerance or modified T_H_2 response [79]. The term modified T_H_2 response stands for a downplay of the downstream effects of normal T_H_2 responses resulting to an increase in non-complement fixing IgG4 and IL-10 [80,81]. In our results, we did not see a significant difference between the IgG4 subclass and the other IgG subclasses amongst patients (as reflected by the OD values (Fig 5)). However, the IgG4 subclass could better differentiate between Mf positive individuals (OVS) and uninfected African controls (HES) as indicated from the ROC curve. This could therefore indicate a bias towards IgG4 in response to OvGM2AP.

In conclusion, during the course of this study, we have established that OvGM2AP (i) is an ESP of *O*. *volvulus*, (ii) is immunogenic and (iii) has characteristics compatible with its use for disease assessment following ivermectin treatment of human onchocerciasis. In this regard, the most important characteristic of OvGM2AP is its ability to discriminate between individuals being treated and those already treated: it therefore holds some potential in certification of disease elimination. Finally, the ongoing elucidation of OvGM2AP function and structure should provide valuable insights into host-parasite interactions as well as better understanding of the nematode biology.

## Supporting information

S1 Table Clinical and demographic data of onchocerciasis subjects. The range, mean and standard deviation (SD) of the age, sex, microfilariae status (mf/skin snip) and observable nodules were assessed for all the investigated onchocerciasis patients (OVS). The percentage of occurrence of symptoms such as pruritus, craw-craw, lymphatic involvement and visual impairment as well as rounds of ivermectin administration were also assessed amongst these patients.
(DOCX)

S2 Table List of hits obtained from OvGM2AP Phyre2 blast. OvGM2AP protein sequence was blasted in the Phyre2 online structure prediction tool and obtained hits were assessed based on confidence and % identity. Confidence reflects the percentage homology between the hit and the OvGM2AP query.
(PDF)

S3 Table Mass spectrometry report for OvGM2AP tryptic digest. (XLSX)

S1 Fig Diagrammatic representation of genomic DNA sequence of OvGM2AP. Genomic DNA (gDNA) sequence of OvGM2AP was analyzed by snap gene and positions of corresponding intron, exons and primers were indicated: **a)** Representation of entire gDNA sequence of OvGM2AP; **b)** Representation of region of amplification.
(TIF)

S2 Fig SDS-PAGE Analysis of 6x His fused OvGM2AP purification from *E. coli*. The protein was expressed in Bl21 cells and purified first by nickel affinity followed by size-exclusion chromatography and analyzed by SDS-PAGE. **(A)** Nickel purification of 6x His fused OvGM2AP. M = Molecular weight marker, 1 = flow through, 2 = wash 1, 3 = wash 2, red arrow indicates elution fraction containing 6x fused OvGM2AP (green arrow) as well as contaminants **(B)** Size–exclusion chromatographic fractions of OvGM2AP. Fraction 15 contains predominantly contaminants while fraction 17 contains predominantly OvGM2AP (green arrow).
(TIF)

S3 Fig Mass spectrometry deamidation peaks of glycosylated peptides. Peptides from tryptic digest of the OvGM2AP were subjected to ion chromatography and the corresponding ion chromatogram generated. Extracted ion chromatogram for two deamidated peptides corresponding to positions 173–183 and 165–177 are indicated with deamidation peaks (red dotted squares) arising from the PNGase F treated sample (Sample B) as opposed to the absence of the peaks in the undigested sample (Sample A).
(TIF)

S4 Fig Analysis of the ROC curve to IgG and IgG subclass responses to OvGM2AP. ELISA Optical density values of infected individuals (OVS) and Control (HES) were used to generate the ROC Curve.
(TIF)

## References

[1] Mawson AR, WaKabongo M. Onchocerciasis-associated morbidity: Hypothesis. Trans R Soc Trop Med Hyg. 2002;96: 541–2. 10.1016/s0035-9203(02)90434-7 12474485

[2] Schulz-Key H, Soboslay PT. Reproductive biology and population dynamics of *Onchocerca volvulus* in the vertebrate host. Parasite. EDP Sciences; 2014;1: S53–S55. 10.1051/parasite/199401s1053

[3] Center for Disease Control C. Parasites—Onchocerciasis (also known as River Blindness). In: Onchocerciasis. 2013.

[4] Organization WH. Progress towards eliminating onchocerciasis in the WHO Region of the Americas: verification of elimination of transmission in Mexico [Internet]. Weekly epidemiological record. 2015 10.1371/jour

[5] WHO. Onchocerciasis (river blindness)—disease information. In: WHO [Internet]. 2017 Available: http://www.who.int/blindness/partnerships/onchocerciasis_disease_information/en/

[6] Twum-Danso NA. *Loa loa* encephalopathy temporally related to ivermectin administration reported from onchocerciasis mass treatment programs from 1989 to 2001: implications for the future. Filaria. 2003;2: S7 10.1186/1475-2883-2-S1-S7 14975064 PMC2147656

[7] Eng JKL, Prichard RK. A comparison of genetic polymorphism in populations of *Onchocerca volvulus* from untreated- and ivermectin-treated patients. Mol Biochem Parasitol. 2005;142: 193–202. 10.1016/j.molbiopara.2005.01.021 15885823

[8] Bourguinat C, Ardelli BF, Pion SDS, Kamgno J, Gardon J, Duke BOL, et al P-glycoprotein-like protein, a possible genetic marker for ivermectin resistance selection in *Onchocerca volvulus*. Mol Biochem Parasitol. 2008;158: 101–111. 10.1016/j.molbiopara.2007.11.017 18215431

[9] Mounchili SC, Ghogomu MA, Ghogomu SM, Njume FN, Robert A. Analysis of *Onchocerca volvulus* β-tubulin gene polymorphism in the Mbonge sub-division of Cameroon: Evidence of gene selection by ivermectin. J Genet Mol Biol. 2018;2: 21–26.

[10] Turner HC, Walker M, Churcher TS, Osei-Atweneboana MY, Biritwum NK, Hopkins A, et al Reaching the London declaration on neglected tropical diseases goals for onchocerciasis: An economic evaluation of increasing the frequency of ivermectin treatment in Africa. Clin Infect Dis. 2014;59: 923–932. 10.1093/cid/ciu467 24944228 PMC4166981

[11] Prichard R, Ménez C, Lespine A. Moxidectin and the avermectins: Consanguinity but not identity. Int J Parasitol Drugs Drug Resist. 2012;2: 134–153. 10.1016/j.ijpddr.2012.04.001 24533275 PMC3862425

[12] Cotreau MM, Warren S, Ryan JL, Fleckenstein L, Vanapalli SR, Brown KR, et al The antiparasitic moxidectin: Safety, tolerability, and pharmacokinetics in humans. J Clin Pharmacol. 2003;43: 1108–1115. 10.1177/0091270003257456 14517193

[13] Korth-Bradley JM, Parks V, Chalon S, Gourley I, Matschke K, Gossart S, et al Excretion of moxidectin into breast milk and pharmacokinetics in healthy lactating women. Antimicrob Agents Chemother. 2011;55: 5200–5204. 10.1128/AAC.00311-11 21896908 PMC3195050

[14] Korth-Bradley JM, Parks V, Chalon S, Gourley I, Matschke K, Cailleux K, et al The effect of a high-fat breakfast on the pharmacokinetics of moxidectin in healthy male subjects: A randomized phase I trial. Am J Trop Med Hyg. 2012;86: 122–125. 10.4269/ajtmh.2012.11-0415 22232462 PMC3247120

[15] Korth-Bradley JM, Parks V, Patat A, Matschke K, Mayer P, Fleckenstein L. Relative Bioavailability of Liquid and Tablet Formulations of the Antiparasitic Moxidectin. Clin Pharmacol Drug Dev. 2012;1: 32–37. 10.1177/2160763X11432508 27206144

[16] Boussinesq M. A new powerful drug to combat river blindness. Lancet. 2018;392: 1170–1172. 10.1016/S0140-6736(18)30101-6 29361336

[17] Weil GJ, Steel C, Liftis F, Li BW, Mearns G, Lobos E, et al A rapid-format antibody card test for diagnosis of onchocerciasis. J Infect Dis. 2000;182: 1796–1799. 10.1086/317629 11069258

[18] Unnasch TR, Golden A, Cama V, Cantey PT. Diagnostics for onchocerciasis in the era of elimination. Int Health. 2018;10: i20–26. 10.1093/inthealth/ihx047 29471336 PMC5881263

[19] Lustigman S, Makepeace BL, Klei TR, Babayan SA, Hotez P, Abraham D, et al *Onchocerca volvulus*: The Road from Basic Biology to a Vaccine. Trends Parasitol. 2018;34: 64–79. 10.1016/j.pt.2017.08.011 28958602 PMC5748257

[20] McNulty SN, Rosa BA, Fischer PU, Rumsey JM, Erdman-Gilmore P, Curtis KC, et al An integrated multi-omics approach to identify candidate antigens for serodiagnosis of human onchocerciasis. Mol Cell Proteomics. 2015;14: 3224–3233. 10.1074/mcp.M115.051953 26472727 PMC4762623

[21] Cotton JA, Bennuru S, Grote A, Harsha B, Tracey A, Beech R, et al The genome of *Onchocerca volvulus*, agent of river blindness. Nat Microbiol. 2016;2: 16216 10.1038/nmicrobiol.2016.216 27869790 PMC5310847

[22] Bennuru S, Cotton JA, Ribeiro JMC, Grote A, Harsha B, Holroyd N, et al Stage-specific transcriptome and proteome analyses of the filarial parasite *Onchocerca volvulus* and its Wolbachia endosymbiont. MBio. 2016;7: e02028–16. 10.1128/mBio.02028-16 27881553 PMC5137501

[23] Shey RA, Ghogomu SM, Njume FN, Gainkam LOT, Poelvoorde P, Mutesa L, et al Prediction and validation of the structural features of Ov58GPCR, an immunogenic determinant of *Onchocerca volvulus*. PLoS One. 2018;13: e0202915 10.1371/journal.pone.0202915 30256790 PMC6157839

[24] Shey RA, Ghogomu SM, Esoh KK, Nebangwa ND, Shintouo CM, Nongley NF, et al In-silico design of a multi-epitope vaccine candidate against onchocerciasis and related filarial diseases. Sci Rep. 2019;9 10.1038/s41598-019-40833-x 30867498 PMC6416346

[25] McKerrow JH, Caffrey C, Kelly B, Loke P, Sajid M. PROTEASES IN PARASITIC DISEASES. Annu Rev Pathol Mech Dis. 2006;1: 497–536. 10.1146/annurev.pathol.1.110304.100151 18039124

[26] Khan AR, Fallon PG. Helminth therapies: Translating the unknown unknowns to known knowns. Int J Parasitol. 2013;43: 293–299. 10.1016/j.ijpara.2012.12.002 23291459

[27] McSorley HJ, Hewitson JP, Maizels RM. Immunomodulation by helminth parasites: Defining mechanisms and mediators. Int J Parasitol. 2013;43: 301–310. 10.1016/j.ijpara.2012.11.011 23291463

[28] Crowe J, Lumb FE, Harnett MM, Harnett W. Parasite excretory-secretory products and their effects on metabolic syndrome. Parasite Immunol. 2017;39: e12410 10.1111/pim.12410 28066896

[29] Boursou D, Ndjonka D, Eisenbarth A, Manchang K, Paguem A, Ngwasiri NN, et al *Onchocerca*—infected cattle produce strong antibody responses to excretory- secretory proteins released from adult male *Onchocerca ochengi* worms. BMC Infect Dis. BMC Infectious Diseases; 2018;18: 1–10. 10.1186/s12879-017-2892-9 29716541 PMC5930424

[30] Eberle R, Brattig NW, Trusch M, Schlüter H, Achukwi MD, Eisenbarth A, et al Isolation, identification and functional profile of excretory-secretory peptides from *Onchocerca ochengi*. Acta Trop. 2015;142: 156–166. 10.1016/j.actatropica.2014.11.015 25479441

[31] Ajonina-Ekoti I, Kurosinski MA, Younis AE, Ndjonka D, Tanyi MK, Achukwi M, et al Comparative analysis of macrophage migration inhibitory factors (MIFs) from the parasitic nematode *Onchocerca volvulus* and the free-living nematode *Caenorhabditis elegans*. Parasitol Res. 2013;112: 3335–46. 10.1007/s00436-013-3513-1 23820606

[32] Borchert N, Becker-Pauly C, Wagner A, Fischer P, Stöcker W, Brattig NW. Identification and characterization of onchoastacin, an astacin-like metalloproteinase from the filaria *Onchocerca volvulus*. Microbes Infect. 2007;9: 498–506. 10.1016/j.micinf.2007.01.007 17347015

[33] Cho-Ngwa F, Zhu X, Metuge JA, Daggfeldt A, Grönvik KO, Orlando R, et al Identification of in vivo released products of *Onchocerca* with diagnostic potential, and characterization of a dominant member, the OV1CF intermediate filament. Infect Genet Evol. 2011;11: 778–83. 10.1016/j.meegid.2010.08.004 20713183

[34] Mackenzie CD, Huntington MK, Wanji S, Lovato R V, Eversole RR, Geary TG. The association of adult *Onchocerca volvulus* with lymphatic vessels. J Parasitol. 2010;96: 219–21. GE-2236 [pii]\r 10.1645/GE-2236.1 19803543

[35] Smith RJ, Cotter TP, Williams JF, Guderian RH. Vascular perfusion of *Onchocerca volvulus* nodules. Trop Med Parasitol. 1988;39: 418–421. 3227242

[36] Meier EM, Schwarzmann G, Fürst W, Sandhoff K. The human GM2 activator protein: A substrate specific cofactor of β-hexosaminidase A. J Biol Chem. 1991;266: 1879–87. 1824846

[37] Mahuran DJ. The GM2 activator protein, its roles as a co-factor in GM2 hydrolysis and as a general glycolipid transport protein. Biochim Biophys Acta—Lipids Lipid Metab. 1998;1393: 1–18. 10.1016/S0005-2760(98)00057-5 9714704

[38] Hama Y, Li YT, Li SC. Interaction of GM2 activator protein with glycosphingolipids. J Biol Chem. 1997;272: 2828–33. 10.1074/jbc.272.5.2828 9006924

[39] Wright CS, Li SC, Rastinejad F. Crystal structure of human GM2-activator protein with a novel β-cup topology. J Mol Biol. 2000;304: 411–22. 10.1006/jmbi.2000.4225 11090283

[40] Wright CS, Zhao Q, Rastinejad F. Structural analysis of lipid complexes of GM2-activator protein. J Mol Biol. 2003;331: 951–964. 10.1016/s0022-2836(03)00794-0 12909021

[41] Kolter T, Sandhoff K. Lysosomal degradation of membrane lipids. FEBS Lett. 2010;584: 1700–12. 10.1016/j.febslet.2009.10.021 19836391

[42] Schulz-Key H. The collagenase technique: how to isolate and examine adult *Onchocerca volvulus* for the evaluation of drug effects. Trop Med Parasitol. 1988;39: 423–440. 2852394

[43] Strote G, Bonow I. Morphological demonstration of essential functional changes after in vitro and in vivo transition of infective *Onchocerca volvulus* to the post-infective stage. Parasitol Res. 1991;77: 526–535. 10.1007/BF00928422 1924261

[44] Cho-Ngwa F, Daggfeldt A, Titanji VPK, Grönvik K-O. Preparation and characterization of specific monoclonal antibodies for the detection of adult worm infections in onchocerciasis. Hybridoma (Larchmt). 2005;24: 283–90. 10.1089/hyb.2005.24.283 16332194

[45] Titanji VPK, Monebenimp F, Ngu JL. Serum immunoglobulin E levels in onchocerciasis: The development of a radioallergosorbent test for *Onchocerca volvulus* infection. Trop Med Parasitol. 1985;36: 12–16. 4001765

[46] Kamga GR, Dissak-Delon FN, Nana-Djeunga HC, Biholong BD, Ghogomu SM, Souopgui J, et al Important progress towards elimination of onchocerciasis in the West Region of Cameroon. Parasites and Vectors. 2017;10: 373 10.1186/s13071-017-2301-7 28774318 PMC5543544

[47] Kamgno J, Nguipdop-Djomo P, Gounoue R, Téjiokem M, Kuesel AC. Effect of Two or Six Doses 800 mg of Albendazole Every Two Months on *Loa loa* Microfilaraemia: A Double Blind, Randomized, Placebo-Controlled Trial. PLoS Negl Trop Dis. 2016;10: e0004492 10.1371/journal.pntd.0004492 26967331 PMC4788450

[48] Gasteiger E., Hoogland C., Gattiker A., Duvaud S., Wilkins M.R., Appel R.D., et al Protein Identification and Analysis Tools on the ExPASy Server The Proteomics Protocols Handbook, Humana Press 2005 pp. 571–607. 10.1385/1592598900

[49] Pei J, Kim BH, Grishin N V. PROMALS3D: A tool for multiple protein sequence and structure alignments. Nucleic Acids Res. 2008;36: 2295–300. 10.1093/nar/gkn072 18287115 PMC2367709

[50] Kelley LA, Mezulis S, Yates CM, Wass MN, Sternberg MJE. The Phyre2 web portal for protein modeling, prediction and analysis. Nat Protoc. 2015;10: 845–58. 10.1038/nprot.2015.053 25950237 PMC5298202

[51] Abdulrahman W, Uhring M, Kolb-Cheynel I, Garnier JM, Moras D, Rochel N, et al A set of baculovirus transfer vectors for screening of affinity tags and parallel expression strategies. Anal Biochem. 2009;385: 383–385. 10.1016/j.ab.2008.10.044 19061853

[52] Osz-Papai J, Radu L, Abdulrahman W, Kolb-Cheynel I, Troffer-Charlier N, Birck C, et al Insect cells-baculovirus system for the production of difficult to express proteins. Insoluble Proteins: Methods and Protocols. 2014 pp. 181–205. 10.1007/978-1-4939-2205-5_10 25447865

[53] Anheuser S, Breiden B, Schwarzmann G, Sandhoff K. Membrane lipids regulate ganglioside GM2 catabolism and GM2 activator protein activity. J Lipid Res. 2015;56: 1747–1761. 10.1194/jlr.M061036 26175473 PMC4548779

[54] Lansmann S, Ferlinz K, Hurwitz R, Bartelsen O, Glombitza G, Sandhoff K. Purification of acid sphingomyelinase from human placenta: Characterization and N-terminal sequence. FEBS Lett. 1996;399: 227–231. 10.1016/s0014-5793(96)01331-2 8985151

[55] Schwarzmann G, Sandhoff K. Lysogangliosides: Synthesis and Use in Preparing Labeled Gangliosides. Methods Enzymol. 1987;138: 319–341. 10.1016/0076-6879(87)38028-0 3600331

[56] MacDonald RC, MacDonald RI, Menco BPM, Takeshita K, Subbarao NK, Hu L rong. Small-volume extrusion apparatus for preparation of large, unilamellar vesicles. BBA—Biomembr. 1991;1061: 297–303. 10.1016/0005-2736(91)90295-J 1998698

[57] Wendeler M, Werth N, Maier T, Schwarzmann G, Kolter T, Schoeniger M, et al The enzyme-binding region of human GM2-activator protein. FEBS J. 2006; 10.1111/j.1742-4658.2006.05126.x 16478472

[58] DeLong ER, DeLong DM, Clarke-Pearson DL. Comparing the Areas under Two or More Correlated Receiver Operating Characteristic Curves: A Nonparametric Approach. Biometrics. 1988;44: 837 10.2307/2531595 3203132

[59] Šimundić A-M. Measures of diagnostic accuracy: Basic definitions. Med Biol Sci. 2008; 1–9. Available: http://www.ifcc.org/ifccfiles/docs/190404200805.pdf PMC4975285 27683318

[60] Titanji V.P.K., Newo A.S., Ghogomu S.M., Souopgui J., Akufongwe P.F., Mbacham W.F. and Fodje M. Titanji, V.P.K, Molecular cloning of OVL3.C1, Marker of Putatively Immunity in Onchocerciasis..pdf. J Cameroon Acad Sci. 2002;2: 285–288.

[61] Wendeler M, Lemm T, Weisgerber J, Hoernschemeyer J, Bartelsen O, Schepers U, et al Expression of recombinant human GM2-activator protein in insect cells: Purification and characterization by mass spectrometry. Protein Expr Purif. 2003;27: 259–66. 10.1016/S1046-5928(02)00599-5 12597885

[62] Kaji H, Kamiie J-I, Kawakami H, Kido K, Yamauchi Y, Shinkawa T, et al Proteomics reveals N-linked glycoprotein diversity in *Caenorhabditis elegans* and suggests an atypical translocation mechanism for integral membrane proteins. Mol Cell Proteomics. 2007;12: 2100–9. 10.1074/mcp.M600392-MCP200 17761667

[63] Wendeler M, Werth N, Maier T, Schwarzmann G, Kolter T, Schoeniger M, et al The enzyme-binding region of human GM2-activator protein. FEBS J. 2006;273: 982–991. 10.1111/j.1742-4658.2006.05126.x 16478472

[64] Conzelmann E, Sandhoff K. Purification and characterization of an activator protein for the degradation of glycolipids GM2 and GA2 by hexosaminidase A. Hoppe Seylers Z Physiol Chem. 1979;360: 1837–49. 10.1515/bchm2.1979.360.2.1837 527942

[65] Bruce AF, Gares MP, Selkirk ME, Gounaris K. Functional characterisation of a nematode secreted GM2-activator protein. Mol Biochem Parasitol. 2006;147: 224–9. 10.1016/j.molbiopara.2006.02.014 16569450

[66] Tropak MB, Yonekawa S, Karumuthil-Melethil S, Thompson P, Wakarchuk W, Gray SJ, et al Construction of a hybrid β-hexosaminidase subunit capable of forming stable homodimers that hydrolyze GM2 ganglioside in vivo. Mol Ther—Methods Clin Dev. Cell Press; 2016;3: 15057 10.1038/mtm.2015.57 26966698 PMC4774620

[67] McDermott M, Wakelam MJ., Morris AJ. Phospholipase D. Biochem Cell Biol. 2004;82: 225–253. 10.1139/o03-079 15052340

[68] Corrotte M, Chasserot-Golaz S, Huang P, Du G, Ktistakis NT, Frohman MA, et al Dynamics and Function of Phospholipase D and Phosphatidic Acid During Phagocytosis. Traffic. 2006;7: 365–377. 10.1111/j.1600-0854.2006.00389.x 16497229

[69] Waselle L, Gerona RRL, Vitale N, Martin TFJ, Bader M-F, Regazzi R. Role of Phosphoinositide Signaling in the Control of Insulin Exocytosis. Mol Endocrinol. 2005;19: 3097–3106. 10.1210/me.2004-0530 16081518

[70] Yang J-S, Gad H, Lee SY, Mironov A, Zhang L, Beznoussenko G V., et al A role for phosphatidic acid in COPI vesicle fission yields insights into Golgi maintenance. Nat Cell Biol. 2008;10: 1146–1153. 10.1038/ncb1774 18776900 PMC2756218

[71] Nishikimi A, Fukuhara H, Su W, Hongu T, Takasuga S, Mihara H, et al Sequential Regulation of DOCK2 Dynamics by Two Phospholipids During Neutrophil Chemotaxis. Science (80-). 2009;324: 384–387. 10.1126/science.1170179 19325080 PMC3761877

[72] Su W, Yeku O, Olepu S, Genna A, Park J-S, Ren H, et al 5-Fluoro-2-indolyl des-chlorohalopemide (FIPI), a Phospholipase D Pharmacological Inhibitor That Alters Cell Spreading and Inhibits Chemotaxis. Mol Pharmacol. 2009;75: 437–446. 10.1124/mol.108.053298 19064628 PMC2684902

[73] Powner DJ, Pettitt TR, Anderson R, Nash GB, Wakelam MJO. Stable adhesion and migration of human neutrophils requires phospholipase D-mediated activation of the integrin CD11b/CD18. Mol Immunol. 2007;44: 3211–3221. 10.1016/j.molimm.2007.01.033 17346796

[74] Klima H, Klein A, van Echten G, Schwarzmann G, Suzuki K, Sandhoff K. Over-expression of a functionally active human GM2-activator protein in *Escherichia coli*. Biochem J. Portland Press Ltd; 1993;292 (Pt 2): 571–6. Available: http://www.ncbi.nlm.nih.gov/pubmed/8503891 8503891 10.1042/bj2920571 PMC1134248

[75] Rigat Brigitte ‡, Wei Wang ‡, Amy Leung ‡ and, Mahuran Don J.* ‡. Two Mechanisms for the Recapture of Extracellular GM2 Activator Protein: Evidence for a Major Secretory Form of the Protein†. Biochemistry. American Chemical Society; 1997;36: 8325–31. 10.1021/BI970571C 9204879

[76] Ran Y, Fanucci GE. Ligand extraction properties of the GM2 activator protein and its interactions with lipid vesicles. Biophys J. 2009;97: 257–266. 10.1016/j.bpj.2009.03.065 19580763 PMC2711380

[77] Koroma JB, Sesay S, Conteh A, Koudou B, Paye J, Bah M, et al Impact of five annual rounds of mass drug administration with ivermectin on onchocerciasis in Sierra Leone. Infect Dis Poverty. 2018;7: 30 10.1186/s40249-018-0410-y 29628019 PMC5890354

[78] Maizels RM, Yazdanbakhsh M. Immune regulation by helminth parasites: cellular and molecular mechanisms. Nat Rev Immunol. 2003;3: 733–744. 10.1038/nri1183 12949497

[79] Rajamanickam Anuradha. Immunomodulation by Filarial Parasites. Int Trends Immun. 2013;1: 12–20. Available: http://researchpub.org/journal/iti/number/vol1-no4/vol1-no4-3.pdf

[80] Adjobimey T, Hoerauf A. Induction of immunoglobulin G4 in human filariasis: an indicator of immunoregulation. Ann Trop Med Parasitol. 2010;104: 455–464. 10.1179/136485910X12786389891407 20863434 PMC3065634

[81] Babu S, Nutman TB. Immunopathogenesis of lymphatic filarial disease. Seminars in Immunopathology. 2012 pp. 847–861. 10.1007/s00281-012-0346-4 23053393 PMC3498535

