# Supplementary material for: Identification and characterization of the Onchocerca volvulus Excretory Secretory Product Ov28CRP, a putative GM2 activator protein
Source: PLoS Negl Trop Dis. 2019 Jul 22;13(7):e0007591. doi: 10.1371/journal.pntd.0007591 (PMC6675134; doi:10.1371/journal.pntd.0007591)
Supplement: S1 Table — The range, mean and standard deviation (SD) of the age, sex, microfilariae status (mf/skin snip) and observable nodules were assessed for all the investigated onchocerciasis patients (OVS). The percentage of occurrence of symptoms such as pruritus, craw-craw, lymphatic involvement and visual impairment as well as rounds of ivermectin administration were also assessed amongst these patients. (DOCX) [file pntd.0007591.s001.docx]

| Age (years) | Range | 10-19 |
| --- | --- | --- |
|  | Mean ± SD | 13.8 ± 2.1 |
| Sex | Males | 19 (63.3 %) |
|  | Females | 11 (36.7 %) |
| Mf/snip | Range | 1-96 |
|  | Mean ± SD | 26.2 ± 28.9 |
| Palpable Nodules | Range | 0-1 |
|  | Mean ± SD | 0.03 ± 0.18 |
| Pruritus | | 20 (66.7 %) |
| Craw-Craw | | 10 (33.3 %) |
| Lymphatic Involvement | | 04 (13.3 %) |
| Visual Impairment | | 1 (3.3 %) |
| Never administered Ivermectin | | 15 ( 50 %) |

**S1 Supplementary table: Clinical and Demographic Data of Onchocerciasis Subjects**

**S1 Supplementary Table: Clinical and Demographic Data of Onchocerciasis Subjects.** The range, mean and standard deviation (SD) of the age, sex, microfilariae status (mf/skin snip) and observable nodules were assessed for all the investigated onchocerciasis patients (OVS). The percentage of occurrence of symptoms such as pruritus, craw-craw, lymphatic involvement and visual impairment as well as rounds of ivermectin administration were also assessed amongst these patients.
