# Supplementary material for: Identification and characterization of the Onchocerca volvulus Excretory Secretory Product Ov28CRP, a putative GM2 activator protein
Source: PLoS Negl Trop Dis. 2019 Jul 22;13(7):e0007591. doi: 10.1371/journal.pntd.0007591 (PMC6675134; doi:10.1371/journal.pntd.0007591)
Supplement: S2 Table — OvGM2AP protein sequence was blasted in the Phyre2 online structure prediction tool and obtained hits were assessed based on confidence and % identity. Confidence reflects the percentage homology between the hit and the OvGM2AP query. (PDF) [file pntd.0007591.s002.pdf]

Phyre2

Email	fngalenjume@gmail.com
Description	OvGM2AP_intensive_modelling
Date	Sat Mar 9 11:18:16 GMT 2019
Unique Job ID	a9c4ebca4c81f6e5

Detailed template information

#	Template	Alignment Coverage	3D Model	Confidence	% i.d.	Template Information
1	d2ag4a1	Alignment	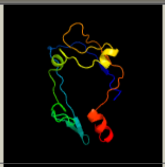	90.0	18	**Fold:**Ganglioside M2 (gm2) activator **Superfamily:**Ganglioside M2 (gm2) activator **Family:**Ganglioside M2 (gm2) activator
2	c3e2uF_	Alignment	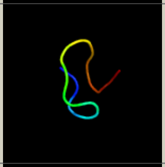	54.6	36	**PDB header:**protein binding **Chain:** F: **PDB Molecule:**cap-gly domain-containing linker protein 1; **PDBTitle:** crystal structure of the zink-knuckle 2 domain of human clip-170 in2 complex with cap-gly domain of human dynactin-1 (p150-glued)
3	c3e2uE_	Alignment	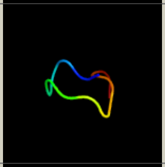	54.6	36	**PDB header:**protein binding **Chain:** E: **PDB Molecule:**cap-gly domain-containing linker protein 1; **PDBTitle:** crystal structure of the zink-knuckle 2 domain of human clip-170 in2 complex with cap-gly domain of human dynactin-1 (p150-glued)
4	c2hqhE_	Alignment	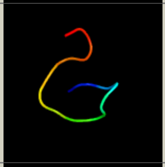	54.6	36	**PDB header:**structural protein, protein binding **Chain:** E: **PDB Molecule:**restin; **PDBTitle:** crystal structure of p150glued and clip-170
5	d1m1ha1	Alignment	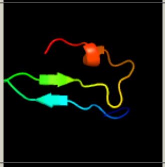	48.3	35	**Fold:**N-utilization substance G protein NusG, insert domain **Superfamily:**N-utilization substance G protein NusG, insert domain **Family:**N-utilization substance G protein NusG, insert domain
6	c5b7iA_	Alignment	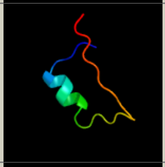	46.3	30	**PDB header:**hydrolase/unknown function **Chain:** A: **PDB Molecule:**crispr-associated nuclease/helicase cas3 subtype i-f/ypest; **PDBTitle:** cas3-acrf3 complex
7	c3e2uH_	Alignment	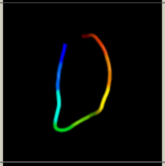	41.3	44	**PDB header:**protein binding **Chain:** H: **PDB Molecule:**cap-gly domain-containing linker protein 1; **PDBTitle:** crystal structure of the zink-knuckle 2 domain of human clip-170 in2 complex with cap-gly domain of human dynactin-1 (p150-glued)
8	c3jb4A_	Alignment	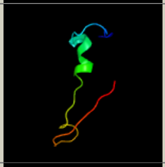	36.7	31	**PDB header:**virus **Chain:** A: **PDB Molecule:**vp1; **PDBTitle:** structure of ljungan virus: insight into picornavirus packaging
9	d1ce7b2	Alignment	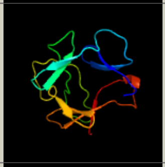	34.1	14	**Fold:**beta-Trefoil **Superfamily:**Ricin B-like lectins **Family:**Ricin B-like
10	c4hspA_	Alignment	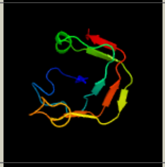	24.2	16	**PDB header:**structural genomics, unknown function **Chain:** A: **PDB Molecule:**hypothetical protein; **PDBTitle:** crystal structure of a duf3299 family protein (pa4066) from2 pseudomonas aeruginosa pao1 at 2.45 a resolution
11	d2a6aa2	Alignment	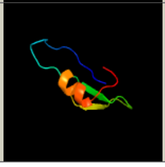	22.7	30	**Fold:**Ribonuclease H-like motif **Superfamily:**Actin-like ATPase domain **Family:**YeaZ-like

12	c2bw2A_	Alignment	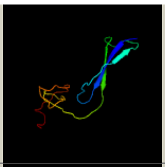	21.6	16	**PDB header:**signaling protein<br>**Chain:** A: **PDB Molecule:**bypass of forespore c;<br>**PDBTitle:** bofc from bacillus subtilis
13	c4qnlA_	Alignment	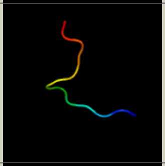	19.5	50	**PDB header:**hydrolase<br>**Chain:** A: **PDB Molecule:**tail fiber protein;<br>**PDBTitle:** crystal structure of tail fiber protein gp63.1 from e. coli phage g7c
14	d1uoza_	Alignment	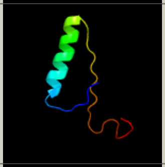	18.3	12	**Fold:**7-stranded beta/alpha barrel<br>**Superfamily:**Glycosyl hydrolases family 6, cellulases<br>**Family:**Glycosyl hydrolases family 6, cellulases
15	d1njha_	Alignment	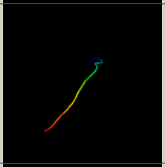	17.6	47	**Fold:**Hypothetical protein YojF<br>**Superfamily:**Hypothetical protein YojF<br>**Family:**Hypothetical protein YojF
16	c4adoA_	Alignment	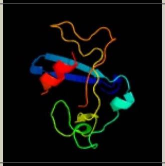	17.1	32	**PDB header:**antibiotic resistance<br>**Chain:** A: **PDB Molecule:**far1;<br>**PDBTitle:** fusidic acid resistance protein fusb
17	d1a9xb2	Alignment	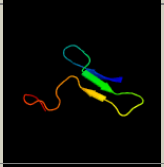	16.4	14	**Fold:**Flavodoxin-like<br>**Superfamily:**Class I glutamine amidotransferase-like<br>**Family:**Class I glutamine amidotransferases (GAT)
18	d1w7ca2	Alignment	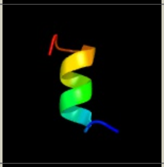	16.0	40	**Fold:**Cystatin-like<br>**Superfamily:**Amine oxidase N-terminal region<br>**Family:**Amine oxidase N-terminal region
19	d1beta_	Alignment	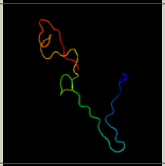	15.2	33	**Fold:**Cystine-knot cytokines<br>**Superfamily:**Cystine-knot cytokines<br>**Family:**Neurotrophin
20	d1vcoa1	Alignment	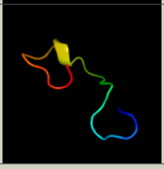	15.2	20	**Fold:**Flavodoxin-like<br>**Superfamily:**Class I glutamine amidotransferase-like<br>**Family:**Class I glutamine amidotransferases (GAT)
21	c6ak0A_	Alignment	not modelled	14.8	62	**PDB header:**unknown function<br>**Chain:** A: **PDB Molecule:**cys-leu-gly-val-gly-ser-cys-val-asp-phe-ala-gly-cys-gly-<br>**PDBTitle:** solution nmr structure of a new lasso peptide specialicin
22	c5tuuB_	Alignment	not modelled	14.8	52	**PDB header:**transcription<br>**Chain:** B: **PDB Molecule:**transcription factor e2f4;<br>**PDBTitle:** crystal structure of the e2f4-dp1 coiled coil and marked-box domains
23	c5yniB_	Alignment	not modelled	14.7	45	**PDB header:**transferase<br>**Chain:** B: **PDB Molecule:**nsp10 protein;<br>**PDBTitle:** crystal structure of mers-cov nsp16/nsp10 complex bound to sam and2 m7gpppg
24	c1jrxA_	Alignment	not modelled	14.7	38	**PDB header:**oxidoreductase<br>**Chain:** A: **PDB Molecule:**flavocytochrome c;<br>**PDBTitle:** crystal structure of arg402ala mutant flavocytochrome c32 from shewanella frigidimarina
25	d1b8mb_	Alignment	not modelled	14.7	30	**Fold:**Cystine-knot cytokines<br>**Superfamily:**Cystine-knot cytokines<br>**Family:**Neurotrophin
26	c6efeA_	Alignment	not modelled	14.7	59	**PDB header:**toxin<br>**Chain:** A: **PDB Molecule:**kappa-conotoxin vil14a;<br>**PDBTitle:** nmr solution structure of vil14a
27	c1qo8A_	Alignment	not modelled	14.5	25	**PDB header:**oxidoreductase<br>**Chain:** A: **PDB Molecule:**flavocytochrome c3 fumarate reductase;<br>**PDBTitle:** the structure of the open conformation of a flavocytochrome2 c3 fumarate reductase
28	d2d8va1	Alignment	not modelled	13.2	42	**Fold:**B-box zinc-binding domain<br>**Superfamily:**B-box zinc-binding domain<br>**Family:**B-box zinc-binding domain
						**PDB header:**transcription

29	c5tuvB_	Alignment	not modelled	12.9	46	**Chain:** B: **PDB Molecule:**transcription factor e2f5;<br>**PDBTitle:** crystal structure of the e2f5-dp1-p107 ternary complex
30	c3wv9D_	Alignment	not modelled	11.9	43	**PDB header:**transferase<br>**Chain:** D: **PDB Molecule:**hmd co-occurring protein hcge;<br>**PDBTitle:** guanylylpyridinol (gp)- and atp-bound hcge from methanothermobacter2 marburgensis
31	c5fu4B_	Alignment	not modelled	11.8	32	**PDB header:**sugar binding protein<br>**Chain:** B: **PDB Molecule:**cbm74-rfgh5;<br>**PDBTitle:** the complexity of the ruminococcus flavefaciens cellulosome reflects2 an expansion in glycan recognition
32	c2d8vA_	Alignment	not modelled	11.3	42	**PDB header:**metal binding protein<br>**Chain:** A: **PDB Molecule:**zinc finger fyve domain-containing protein 19;<br>**PDBTitle:** solution structure of the b-box domain of the zinc finger2 fyve domain-containing protein 19 from mus musculus
33	c3fptC_	Alignment	not modelled	11.0	43	**PDB header:**immune system<br>**Chain:** C: **PDB Molecule:**evasin-1;<br>**PDBTitle:** the crystal structure of the complex between evasin-1 and2 ccl3
34	c3ij2B_	Alignment	not modelled	11.0	28	**PDB header:**hormone/protein binding<br>**Chain:** B: **PDB Molecule:**beta-nerve growth factor;<br>**PDBTitle:** ligand-receptor structure
35	d2bodx1	Alignment	not modelled	10.9	17	**Fold:**7-stranded beta/alpha barrel<br>**Superfamily:**Glycosyl hydrolases family 6, cellulases<br>**Family:**Glycosyl hydrolases family 6, cellulases
36	d2aaib2	Alignment	not modelled	10.7	13	**Fold:**beta-Trefoil<br>**Superfamily:**Ricin B-like lectins<br>**Family:**Ricin B-like
37	c1keeH_	Alignment	not modelled	10.5	15	**PDB header:**ligase<br>**Chain:** H: **PDB Molecule:**carbamoyl-phosphate synthetase small chain;<br>**PDBTitle:** inactivation of the amidotransferase activity of carbamoyl phosphate2 synthetase by the antibiotic acivicin
38	c2g9tT_	Alignment	not modelled	10.5	39	**PDB header:**viral protein<br>**Chain:** T: **PDB Molecule:**<br>**PDBTitle:** crystal structure of the sars coronavirus nsp10 at 2.1a
39	c6faoA_	Alignment	not modelled	10.5	24	**PDB header:**hydrolase<br>**Chain:** A: **PDB Molecule:**glycoside hydrolase family 6;<br>**PDBTitle:** discovery and characterization of a thermostable gh6 endoglucanase2 from a compost metagenome
40	c4d7aA_	Alignment	not modelled	10.4	86	**PDB header:**ligase<br>**Chain:** A: **PDB Molecule:**trna threonylcarbamoyladenosine dehydratase;<br>**PDBTitle:** crystal structure of e. coli trna n6-threonylcarbamoyladenosine2 dehydratase, tcda, in complex with amp at 1.801 angstroem3 resolution
41	c1rpbA_	Alignment	not modelled	10.4	71	**PDB header:**replication inhibitor<br>**Chain:** A: **PDB Molecule:**tricyclic peptide rp 71955;<br>**PDBTitle:** solution structure of rp 71955, a new 21 amino acid tricyclic peptide2 active against hiv-1 virus
42	d1xjha_	Alignment	not modelled	10.4	17	**Fold:**HSP33 redox switch-like<br>**Superfamily:**HSP33 redox switch-like<br>**Family:**HSP33 redox switch-like
43	d2fyga1	Alignment	not modelled	10.3	39	**Fold:**Coronavirus NSP10-like<br>**Superfamily:**Coronavirus NSP10-like<br>**Family:**Coronavirus NSP10-like
44	d1vzya2	Alignment	not modelled	10.1	30	**Fold:**HSP33 redox switch-like<br>**Superfamily:**HSP33 redox switch-like<br>**Family:**HSP33 redox switch-like
45	c1rpcA_	Alignment	not modelled	9.9	71	**PDB header:**replication inhibitor<br>**Chain:** A: **PDB Molecule:**tricyclic peptide rp 71955;<br>**PDBTitle:** solution structure of rp 71955, a new 21 amino acid tricyclic peptide2 active against hiv-1 virus
46	d1vm8a_	Alignment	not modelled	9.7	24	**Fold:**Nucleotide-diphospho-sugar transferases<br>**Superfamily:**Nucleotide-diphospho-sugar transferases<br>**Family:**UDP-glucose pyrophosphorylase
47	c2ro1A_	Alignment	not modelled	9.5	20	**PDB header:**transcription<br>**Chain:** A: **PDB Molecule:**transcription intermediary factor 1-beta;<br>**PDBTitle:** nmr solution structures of human kap1 phd finger-bromodomain
48	c2mekA_	Alignment	not modelled	9.3	21	**PDB header:**metal binding protein<br>**Chain:** A: **PDB Molecule:**n-terminal domain of bilbo1;<br>**PDBTitle:** n-terminal domain of bilbo1 from trypanosoma brucei
49	d1hcfa_	Alignment	not modelled	9.1	33	**Fold:**Cystine-knot cytokines<br>**Superfamily:**Cystine-knot cytokines<br>**Family:**Neurotrophin
50	c3p1mG_	Alignment	not modelled	9.0	33	**PDB header:**electron transport<br>**Chain:** G: **PDB Molecule:**adrenodoxin, mitochondrial;<br>**PDBTitle:** crystal structure of human ferredoxin-1 (fdx1) in complex with iron-2 sulfur cluster
51	c2vxoB_	Alignment	not modelled	8.8	30	**PDB header:**ligase<br>**Chain:** B: **PDB Molecule:**gmp synthase [glutamine-hydrolyzing];<br>**PDBTitle:** human gmp synthetase in complex with xmp
52	c4pwuC_	Alignment	not modelled	8.8	40	**PDB header:**signaling protein<br>**Chain:** C: **PDB Molecule:**modulator protein mzra;<br>**PDBTitle:** crystal structure of a modulator protein mzra (kpn_03524) from2 klebsiella pneumoniae subsp. pneumoniae mgh 78578 at 2.45 a3 resolution
53	c3higB_	Alignment	not modelled	8.8	38	**PDB header:**oxidoreductase<br>**Chain:** B: **PDB Molecule:**amiloride-sensitive amine oxidase;<br>**PDBTitle:** crystal structure of human diamine oxidase in complex with the2 inhibitor berenil
54	c3uowB_	Alignment	not modelled	8.8	26	**PDB header:**ligase<br>**Chain:** B: **PDB Molecule:**gmp synthetase;<br>**PDBTitle:** crystal structure of pf10_0123, a gmp synthetase from

						plasmodium2 falciparum
55	c3gucB_	Alignment	not modelled	8.6	86	**PDB header:**transferase<br>**Chain:** B: **PDB Molecule:**ubiquitin-like modifier-activating enzyme 5;<br>**PDBTitle:** human ubiquitin-activating enzyme 5 in complex with amppnp
56	c3lxfC_	Alignment	not modelled	8.6	50	**PDB header:**metal binding protein<br>**Chain:** C: **PDB Molecule:**ferredoxin;<br>**PDBTitle:** crystal structure of [2fe-2s] ferredoxin arx from novosphingobium2 aromaticivorans
57	c4lu2A_	Alignment	not modelled	8.5	33	**PDB header:**odorant-binding protein<br>**Chain:** A: **PDB Molecule:**10.7 kda salivary protein;<br>**PDBTitle:** structure of ljm19
58	c3cnfT_	Alignment	not modelled	8.5	47	**PDB header:**virus<br>**Chain:** T: **PDB Molecule:**vp3;<br>**PDBTitle:** 3.88 angstrom structure of cytoplasmic polyhedrosis virus2 by cryo-electron microscopy
59	c6h77B_	Alignment	not modelled	8.5	39	**PDB header:**signaling protein<br>**Chain:** B: **PDB Molecule:**ubiquitin-like modifier-activating enzyme 5;<br>**PDBTitle:** e1 enzyme for ubiquitin like protein activation in complex with ubl
60	d1bkba1	Alignment	not modelled	8.5	29	**Fold:**SH3-like barrel<br>**Superfamily:**Translation proteins SH3-like domain<br>**Family:**eIF5a N-terminal domain-like
61	d2i1sa1	Alignment	not modelled	8.4	17	**Fold:**MM3350-like<br>**Superfamily:**MM3350-like<br>**Family:**MM3350-like
62	d1e9ma_	Alignment	not modelled	8.4	32	**Fold:**beta-Grasp (ubiquitin-like)<br>**Superfamily:**2Fe-2S ferredoxin-like<br>**Family:**2Fe-2S ferredoxin-related
63	d1y88a1	Alignment	not modelled	8.3	45	**Fold:**SAM domain-like<br>**Superfamily:**Rad51 N-terminal domain-like<br>**Family:**Hypothetical protein AF1548, C-terminal domain
64	c4hceA_	Alignment	not modelled	8.2	55	**PDB header:**cell cycle<br>**Chain:** A: **PDB Molecule:**cell division control protein 13;<br>**PDBTitle:** crystal structure of the telomeric saccharomyces cerevisiae cdc13 ob22 domain
65	c5fu7D_	Alignment	not modelled	8.2	50	**PDB header:**gene regulation<br>**Chain:** D: **PDB Molecule:**nanos, isoform b;<br>**PDBTitle:** drosophila nanos nbr peptide bound to the not module2 of the human ccr4-not complex
66	c1h32A_	Alignment	not modelled	8.2	11	**PDB header:**electron transfer<br>**Chain:** A: **PDB Molecule:**diheme cytochrome c;<br>**PDBTitle:** reduced soxax complex from rhodovulum sulfidophilum
67	c4owlA_	Alignment	not modelled	8.1	15	**PDB header:**toxin<br>**Chain:** A: **PDB Molecule:**cytolysin;<br>**PDBTitle:** crystal structure of the vibrio vulnificus hemolysin/cytolysin beta-2 trefoil lectin with n-acetyl-d-lactosamine bound
68	c1gpmD_	Alignment	not modelled	8.1	22	**PDB header:**transferase (glutamine amidotransferase)<br>**Chain:** D: **PDB Molecule:**gmp synthetase;<br>**PDBTitle:** escherichia coli gmp synthetase complexed with amp and pyrophosphate
69	c2ywjA_	Alignment	not modelled	8.0	36	**PDB header:**transferase<br>**Chain:** A: **PDB Molecule:**glutamine amidotransferase subunit pdxt;<br>**PDBTitle:** crystal structure of uncharacterized conserved protein from2 methanocaldococcus jannaschii
70	c1zfnA_	Alignment	not modelled	7.9	57	**PDB header:**transferase<br>**Chain:** A: **PDB Molecule:**adenylyltransferase thif;<br>**PDBTitle:** structural analysis of escherichia coli thif
71	c5vj7B_	Alignment	not modelled	7.9	23	**PDB header:**oxidoreductase<br>**Chain:** B: **PDB Molecule:**ferredoxin-nadp(+) reductase subunit alpha;<br>**PDBTitle:** ferredoxin nadp oxidoreductase (xfn)
72	c5cl2A_	Alignment	not modelled	7.9	17	**PDB header:**protein binding<br>**Chain:** A: **PDB Molecule:**sporulation-control protein spo0m;<br>**PDBTitle:** crystal structure of spo0m, sporulation control protein, from bacillus2 subtilis.
73	c5ts4A_	Alignment	not modelled	7.8	29	**PDB header:**de novo protein<br>**Chain:** A: **PDB Molecule:**denovo ntf2;<br>**PDBTitle:** crystal structure of a de novo designed protein with curved beta-sheet
74	c1x4iA_	Alignment	not modelled	7.8	53	**PDB header:**cell adhesion<br>**Chain:** A: **PDB Molecule:**inhibitor of growth protein 3;<br>**PDBTitle:** solution structure of phd domain in inhibitor of growth2 protein 3 (ing3)
75	c3mw3A_	Alignment	not modelled	7.7	12	**PDB header:**cell adhesion<br>**Chain:** A: **PDB Molecule:**neurexin-2-beta;<br>**PDBTitle:** crystal structure of beta-neurexin 2 with the splice insert 4
76	c4bmaB_	Alignment	not modelled	7.6	29	**PDB header:**transferase<br>**Chain:** B: **PDB Molecule:**udp-n-acetylglucosamine pyrophosphorylase;<br>**PDBTitle:** structural of aspergillus fumigatus udp-n-acetylglucosamine2 pyrophosphorylase
77	c3tqiB_	Alignment	not modelled	7.6	22	**PDB header:**ligase<br>**Chain:** B: **PDB Molecule:**gmp synthase [glutamine-hydrolyzing];<br>**PDBTitle:** structure of the gmp synthase (guaa) from coxiella burnetii
78	c3a9fA_	Alignment	not modelled	7.5	22	**PDB header:**electron transport<br>**Chain:** A: **PDB Molecule:**cytochrome c;<br>**PDBTitle:** crystal structure of the c-terminal domain of cytochrome cz from2 chlorobium tepidum
79	c1w7cA_	Alignment	not modelled	7.4	31	**PDB header:**oxidoreductase<br>**Chain:** A: **PDB Molecule:**lysyl oxidase;<br>**PDBTitle:** pplo at 1.23 angstroms

80	c3u6gB_	Alignment	not modelled	7.4	31	**PDB header:**structural genomics, unknown function **Chain:** B: **PDB Molecule:**uncharacterized protein duf4425; **PDBTitle:** crystal structure of a domain of unknown function, duf4425 (bvu_3708)2 from bacteroides vulgatus atcc 8482 at 1.35 a resolution
81	c4divH_	Alignment	not modelled	7.2	22	**PDB header:**viral protein **Chain:** H: **PDB Molecule:**orf48; **PDBTitle:** the structure of a 1.8 mda viral genome injection device suggests2 alternative infection mechanisms
82	d1qjta_	Alignment	not modelled	7.2	16	**Fold:**EF Hand-like **Superfamily:**EF-hand **Family:**Eps15 homology domain (EH domain)
83	c1lf6A_	Alignment	not modelled	7.2	33	**PDB header:**hydrolase **Chain:** A: **PDB Molecule:**glucoamylase; **PDBTitle:** crystal structure of bacterial glucoamylase
84	c3h9gA_	Alignment	not modelled	7.1	67	**PDB header:**transferase/antibiotic **Chain:** A: **PDB Molecule:**mccb protein; **PDBTitle:** crystal structure of e. coli mccb + mcca-n7isoasn
85	d1b9ra_	Alignment	not modelled	7.1	37	**Fold:**beta-Grasp (ubiquitin-like) **Superfamily:**2Fe-2S ferredoxin-like **Family:**2Fe-2S ferredoxin-related
86	c1n9eA_	Alignment	not modelled	7.0	31	**PDB header:**oxidoreductase **Chain:** A: **PDB Molecule:**lysyl oxidase; **PDBTitle:** crystal structure of pichia pastoris lysyl oxidase pplo
87	d1jv1a_	Alignment	not modelled	7.0	24	**Fold:**Nucleotide-diphospho-sugar transferases **Superfamily:**Nucleotide-diphospho-sugar transferases **Family:**UDP-glucose pyrophosphorylase
88	c1vzyA_	Alignment	not modelled	7.0	30	**PDB header:**chaperone **Chain:** A: **PDB Molecule:**33 kda chaperonin; **PDBTitle:** crystal structure of the bacillus subtilis hsp33
89	d1s16a1	Alignment	not modelled	6.9	32	**Fold:**Ribosomal protein S5 domain 2-like **Superfamily:**Ribosomal protein S5 domain 2-like **Family:**DNA gyrase/MutL, second domain
90	c4ltuB_	Alignment	not modelled	6.9	26	**PDB header:**electron transport **Chain:** B: **PDB Molecule:**ferredoxin; **PDBTitle:** crystal structure of ferredoxin from rhodopseudomonas palustris haa2
91	c5zwoW_	Alignment	not modelled	6.8	50	**PDB header:**splicing **Chain:** W: **PDB Molecule:**23 kda u4/u6.u5 small nuclear ribonucleoprotein component; **PDBTitle:** cryo-em structure of the yeast b complex at average resolution of 3.92 angstrom
92	c2n99A_	Alignment	not modelled	6.8	38	**PDB header:**neuropeptide **Chain:** A: **PDB Molecule:**ly-6/neurotoxin-like protein 1; **PDBTitle:** solution structure of the slurp-2, a secreted isoform of lynx1
93	c3jtcD_	Alignment	not modelled	6.7	41	**PDB header:**blood clotting **Chain:** D: **PDB Molecule:**vitamin k-dependent protein c; **PDBTitle:** importance of mg2+ in the ca2+-dependent folding of the gamma-2 carboxyglutamic acid domains of vitamin k-dependent clotting and3 anticlotting proteins
94	c6az5A_	Alignment	not modelled	6.7	42	**PDB header:**sugar binding protein **Chain:** A: **PDB Molecule:**alpha-amylase; **PDBTitle:** crystal structure of cbmd (family cbm41) from eubacterium rectale2 amy13k
95	c5o9zN_	Alignment	not modelled	6.6	50	**PDB header:**splicing **Chain:** N: **PDB Molecule:**zinc finger matrin-type protein 2; **PDBTitle:** cryo-em structure of a pre-catalytic human spliceosome primed for2 activation (b complex)
96	c2ywcC_	Alignment	not modelled	6.6	38	**PDB header:**ligase **Chain:** C: **PDB Molecule:**gmp synthase [glutamine-hydrolyzing]; **PDBTitle:** crystal structure of gmp synthetase from thermus thermophilus in2 complex with xmp
97	d1xx6a2	Alignment	not modelled	6.5	19	**Fold:**Glucocorticoid receptor-like (DNA-binding domain) **Superfamily:**Glucocorticoid receptor-like (DNA-binding domain) **Family:**Type II thymidine kinase zinc finger
98	c3jtcC_	Alignment	not modelled	6.5	41	**PDB header:**blood clotting **Chain:** C: **PDB Molecule:**vitamin k-dependent protein c; **PDBTitle:** importance of mg2+ in the ca2+-dependent folding of the gamma-2 carboxyglutamic acid domains of vitamin k-dependent clotting and3 anticlotting proteins
99	c1lqvD_	Alignment	not modelled	6.5	41	**PDB header:**blood clotting **Chain:** D: **PDB Molecule:**vitamin-k dependent protein c; **PDBTitle:** crystal structure of the endothelial protein c receptor with2 phospholipid in the groove in complex with gla domain of protein c.
