# Supplementary figures and images for: Identification and characterization of the Onchocerca volvulus Excretory Secretory Product Ov28CRP, a putative GM2 activator protein
Source: PLoS Negl Trop Dis. 2019 Jul 22;13(7):e0007591. doi: 10.1371/journal.pntd.0007591 (PMC6675134; doi:10.1371/journal.pntd.0007591)

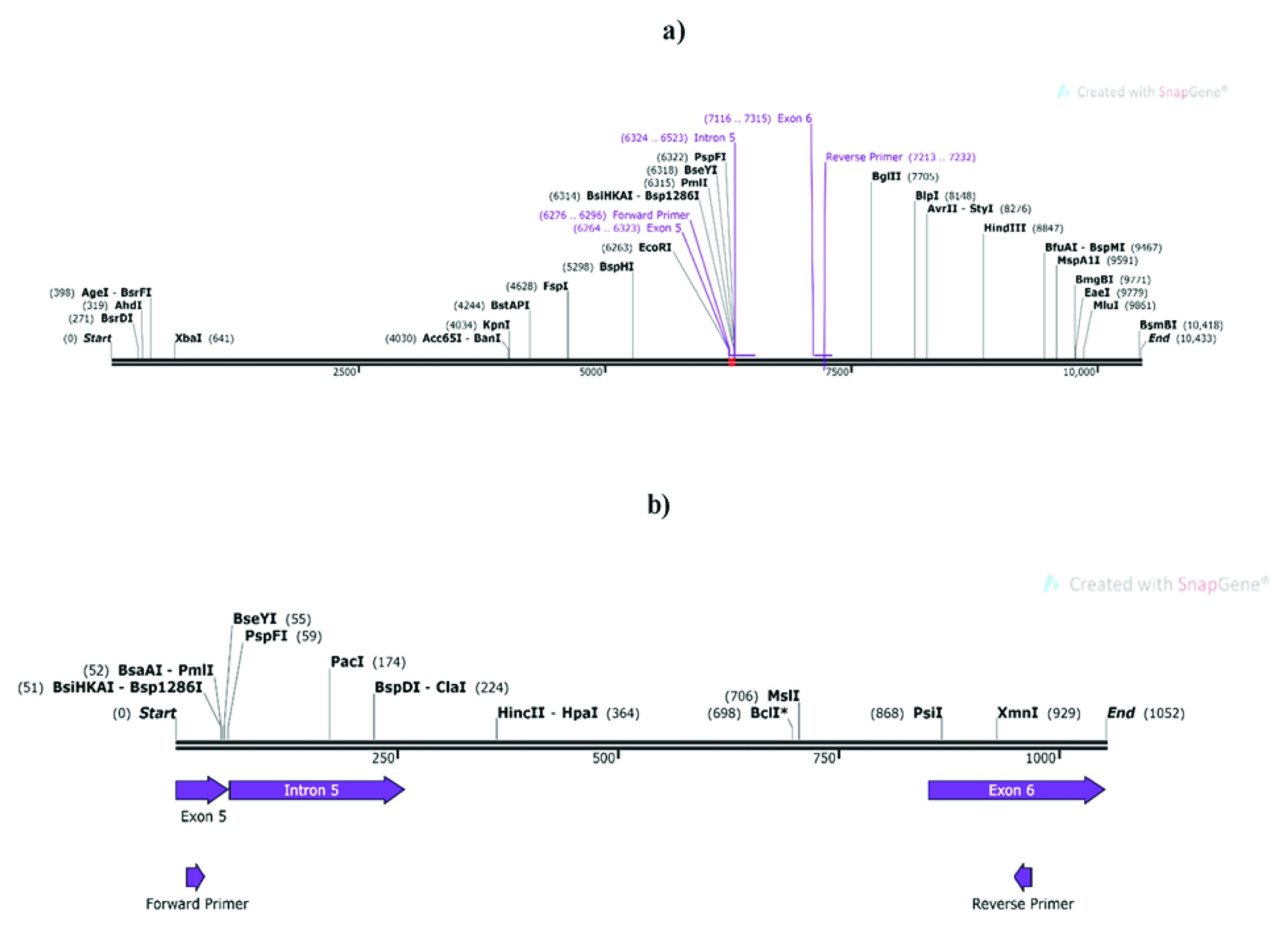

Supplement: S1 Fig — Genomic DNA (gDNA) sequence of OvGM2AP was analyzed by snap gene and positions of corresponding intron, exons and primers were indicated: a) Representation of entire gDNA sequence of OvGM2AP; b) Representation of region of amplification. (TIF) [file pntd.0007591.s004.tif]

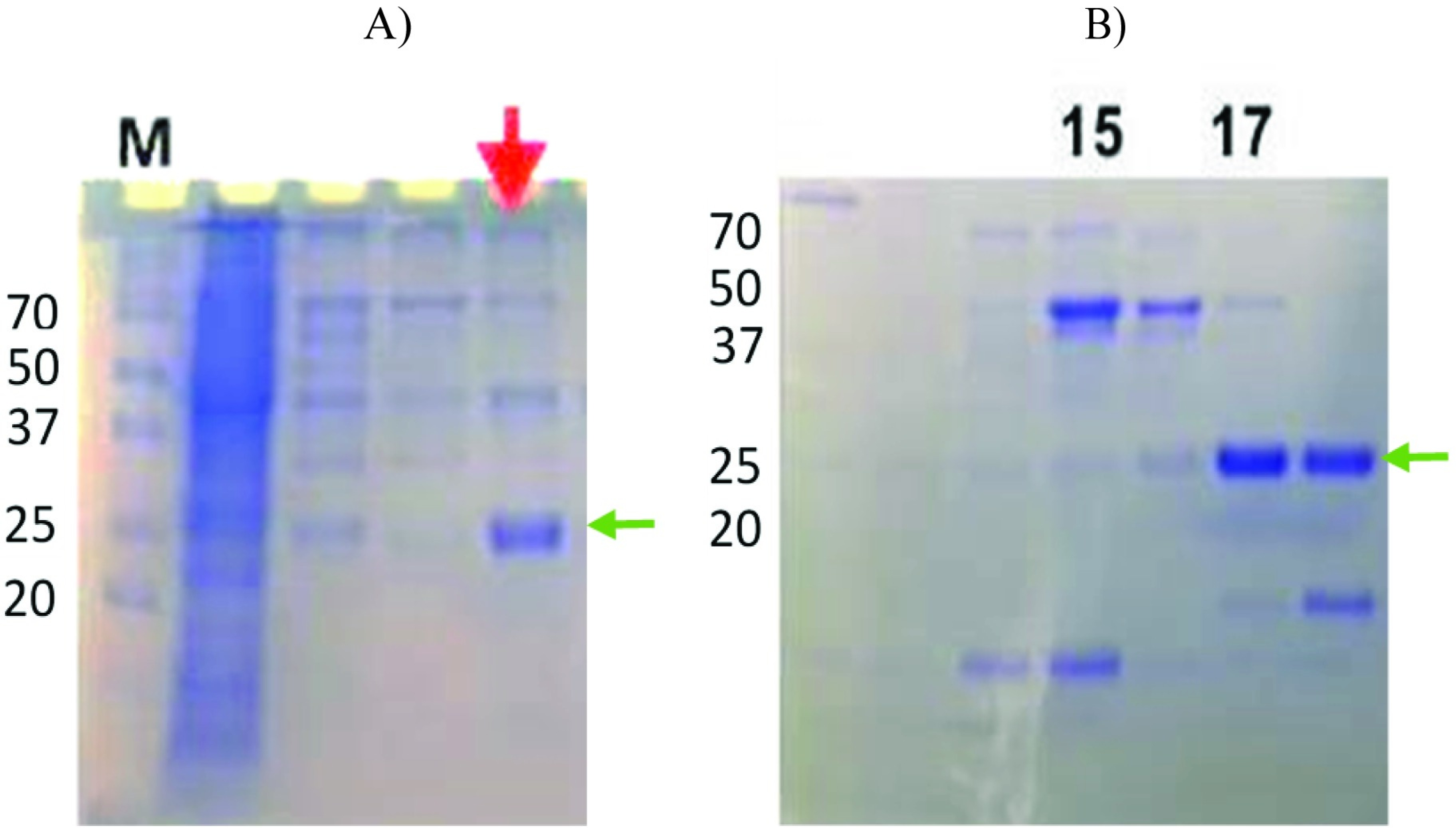

Supplement: S2 Fig — The protein was expressed in Bl21 cells and purified first by nickel affinity followed by size-exclusion chromatography and analyzed by SDS-PAGE. (A) Nickel purification of 6x His fused OvGM2AP. M = Molecular weight marker, 1 = flow through, 2 = wash 1, 3 = wash 2, red arrow indicates elution fraction containing 6x fused OvGM2AP (green arrow) as well as contaminants (B) Size–exclusion chromatographic fractions of OvGM2AP. Fraction 15 contains predominantly contaminants while fraction 17 contains predominantly OvGM2AP (green arrow). (TIF) [file pntd.0007591.s005.tif]

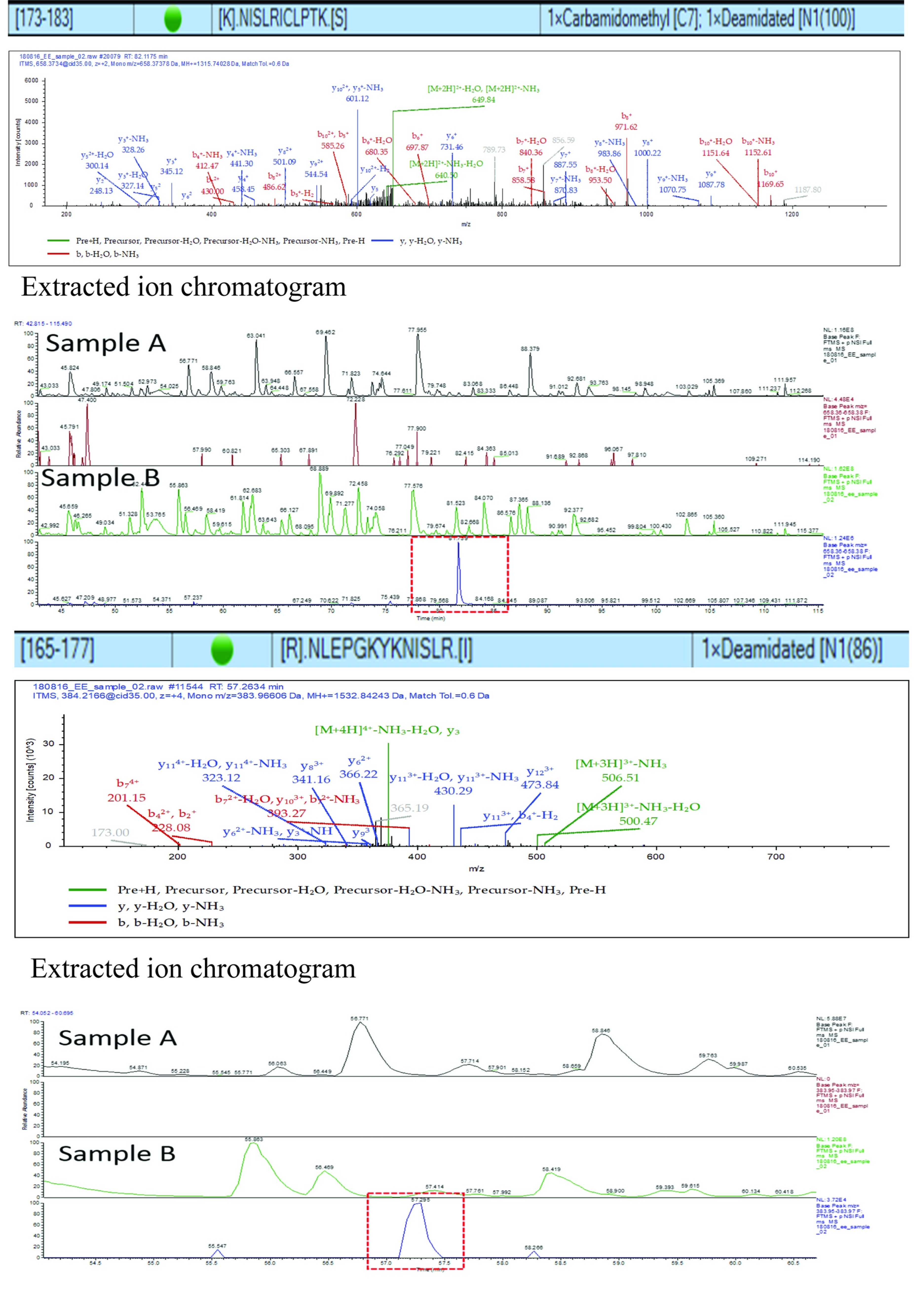

Supplement: S3 Fig — Peptides from tryptic digest of the OvGM2AP were subjected to ion chromatography and the corresponding ion chromatogram generated. Extracted ion chromatogram for two deamidated peptides corresponding to positions 173–183 and 165–177 are indicated with deamidation peaks (red dotted squares) arising from the PNGase F treated sample (Sample B) as opposed to the absence of the peaks in the undigested sample (Sample A). (TIF) [file pntd.0007591.s006.tif]

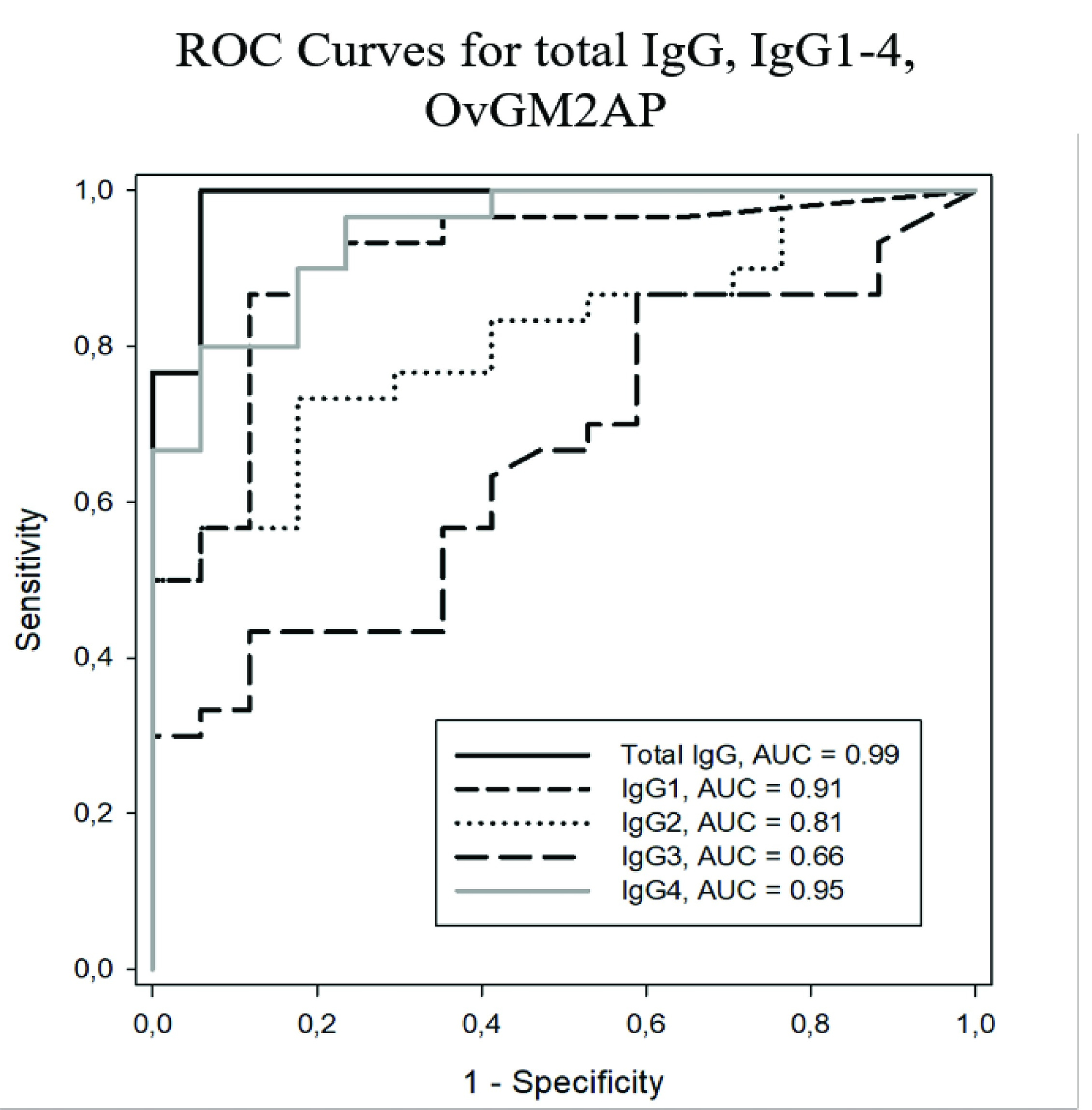

Supplement: S4 Fig — ELISA Optical density values of infected individuals (OVS) and Control (HES) were used to generate the ROC Curve. (TIF) [file pntd.0007591.s007.tif]
